# The majority of Norwegian patients with treatment-resistant chronic pain regained normal national health standards within 12 months after De-Qi acupuncture - a prospective observational propensity score matched study

**DOI:** 10.3389/fpain.2025.1521466

**Published:** 2025-04-08

**Authors:** Veronika Lindberg, Jan Baak

**Affiliations:** ^1^ Department of Statistics, Lintech AS, Kristiansand, Norway; ^2^ Department of Quantitative and Molecular Pathology, Stavanger University Hospital, Stavanger, Norway

**Keywords:** acupuncture, De-Qi, chronic pain, theraphy resistant, family practice, full remission, propensity score analysis (PSA), health-related quality of life (HRQL)

## Abstract

**Purpose:**

In previous studies, acupuncture was effective in the treatment of patients with chronic pain that was unresponsive to conventional therapies. However, the proportion of patients in a real-world setting who regain normal health after 1 year, following De-Qi acupuncture is unknown.

**Methods:**

This is an observational prospective study of 354 new patients in a family medical practice between 2015 and 2018. Patients self-assessed pain using the Visual Analogue Scale (VAS), and health using the Short Form 36 Health Survey (SF-36) before treatment and at 3- and 12-months of follow-up. VAS and SF-36 components were compared for improvements and therapeutic effect sizes. Propensity score matching was employed to avoid bias by confounding variables.

**Results:**

The participation rate was 29%, median age 50 years (range 20–79), 65% were females, median pain duration was 18 months (6–360), the median number of acupuncture treatments was 6 (1–27). The initial VAS pain intensity of 6.2 (SD 2.5) improved to 4.0 at 3 months and 3.2 at 12 months (*p* < .001, large effect size); SF-36 scores also improved. 75% of patients showed strong responses, with 58% reaching complete cure and 17% achieving near-normal health. Patients aged >65 responded well and ≥6 treatments were associated with stronger responses than 1–5. Hill criteria analysis of improvements with acupuncture suggested causation over association.

**Conclusion:**

In patients with chronic pain (median 18 months), who were unresponsive to conventional treatments, De-Qi acupuncture was associated with sustained pain reduction and health improvements. Most attained Norwegian national normal health standards (complete cure) after 12-months.

## Background

Chronic pain (≥3 months) is a very frequent condition that is treated primarily by first-line medical professionals (1). Chronic pain is particularly prevalent among those over 65 years of age and is associated with accelerated cognitive decline and premature death (2), affects daily work and life activities and is linked to depression (3), suicide risk (4), and drug misuse (5). 21% of U.S. adults experience chronic pain and the annual costs of healthcare and decreased work productivity is 560–635 billion US dollars (6). Chronic pain prevalence among adults in Europe (7) and in Norway can be as high as 30% (8). Very many Norwegian chronic pain patients were long-term opioid users (9).

Prospective clinical trials on pain have shown a strong positive effect of acupuncture (10–12). The World Health Organization, in its 2018 International Classification of Diseases, 11th Revision (ICD-11), included Traditional Chinese Medicine (TCM) in its classification system (13). Nevertheless, skepticism toward acupuncture among medical doctors is not unusual and very few doctors in Norway use acupuncture. This is concerning, because a recent Norwegian analysis found that 61% of the general population and 89% of cancer patients used Complementary and Alternative Medicine in the 12 months prior to the study (14). Due to this skepticism and the limited adoption of acupuncture in clinical practice, many medical doctors remain unaware that acupuncture can effectively reduce chronic pain and, in some cases, eliminate it completely in real-world settings. While clinical trials on acupuncture exist, there has been limited investigation of its effects in prospective real-world general medical practice settings.

Given this gap in real-world evidence, we conducted a prospective observational study to assess the long-term therapeutic efficacy of acupuncture in chronic pain patients unresponsive to conventional treatments. The study included all new patients with chronic pain (>3 months duration) treated in our medical practice between 2015 and 2017, with a one-year follow-up.

In TCM, the therapeutic effect of acupuncture is believed to be closely linked to the achievement of “De-Qi” (obtaining Qi or Qi arrival), a distinct sensation that signifies the activation of Qi flow in the meridians. This sensation is traditionally considered essential for acupuncture's clinical efficacy and is a key factor in treatment outcomes (15–17). It is important to emphasize that achieving De-Qi sensations was a key objective during all treatments in this study. As this concept is not universally well-known or consistently practiced among acupuncturists, a brief explanation follows.

De-Qi was first described in the Huang Di Nei Jing (475–221 BC) and refers to specific sensations experienced by patients when an acupuncture needle is properly inserted into an acupuncture point. These sensations may include numbness, tingling, swelling, heaviness, pain, heat, or cold, often radiating locally or peripherally. Simultaneously, practitioners may perceive tremors in the needle or a sudden increase in resistance, sometimes described as a “fish-bite” sensation, indicating proper engagement with the meridian. De-Qi is considered a response to Qi (vital energy) movement within the affected meridian, contrasting with the nearly painless insertion of needles in non-meridian areas, where no acupuncture points exist (18–20).

An excellent review on De-Qi is available (21). Over recent decades, debate has emerged regarding whether acupuncture without De-Qi has any therapeutic effect. Recent research has demonstrated that De-Qi is a critical factor in achieving optimal therapeutic efficacy (22). In clinical practice, De-Qi may therefore be a crucial variable in studies investigating the mechanism and effectiveness of acupuncture treatment (17).

Another important methodological aspect of the current study relates to statistical analysis. While randomized clinical trials are considered the gold standard for reliable results, our approach to acupuncture presents unique challenges. Since we aim to achieve De-Qi during all treatments, a sensation that patients clearly feel, we cannot use control points without known therapeutic effect, as these points do not typically produce De-Qi sensations. Therefore, we analyzed treatment outcomes without a traditional control group, specifically examining the rates of “cure” or “significant clinical improvement” across our patient population.

Such prospective observational studies carry an inherent risk that patient outcomes could be influenced by confounders (non-treatment baseline features that affect prognosis). Without controlling for these potential confounders, treatment effects may appear overly optimistic. To address this limitation, we employed propensity score matching (PSM), a statistical method that has gained widespread acceptance in observational research over the past decades (23, 24). Although PSM can effectively overcome many concerns related to confounding variables, it has not been widely applied in acupuncture observational studies. We utilized this approach in our prospective study conducted in a family medicine practice, evaluating the therapeutic effects of De-Qi acupuncture on new patients with therapy-resistant chronic pain from 2015 to 2018.

It is important to emphasize that our study population consisted exclusively of patients who had been deemed untreatable by other medical doctors and practitioners (including physiotherapists and chiropractors) after conventional therapies proved unsuccessful. This means our patient group presented with a prognostically unfavorable profile from the outset, making any positive outcomes particularly noteworthy.

We also introduced a novel statistical approach to evaluate acupuncture success by quantifying the proportion of patients who regained normal health according to Norwegian national health standards. Additionally, we applied the Bradford Hill criteria to assess whether the relationship between De-Qi acupuncture and observed health improvements was causal rather than merely associative (25, 26). This comprehensive evaluation framework strengthens the interpretation of our findings beyond traditional outcome measures used in acupuncture research.

## Methods

This was a non-interventional study because medicines were prescribed in the usual manner, as stipulated by marketing authorizations. This was also an observational study because the effects of risk factors, treatments, or other interventions were not controlled (27).

Ethical approval was granted by the Norwegian Social Science Data Services (project 37235, 2014/02/21). The initial enrollment span (q4 2015–q4 2016) was extended to the end of q4 2018, for which additional ethical approval was obtained from the Norwegian Data Protection Authority (Datatilsynet, 2017/05/22).

All consecutive new patients aged 18 years or older, who were able to read, write, and understand Norwegian, and who visited the outpatient family medicine practice of Dr. med. Jan Baak AS, Tananger, Norway, from October 2015 to December 2017 for any disease condition, with follow-up until December 2018, were considered for enrollment. Specific inclusion criteria for this study were: (1) chronic pain defined as persistent or recurrent pain lasting for at least 3 months; (2) pain located in the neck, shoulder, back, hip, or knee regions identified by ICD-11 codes; (3) previous unsuccessful treatments with conventional therapies (such as pain medications, corticosteroid injections, physical therapy and exercise); and (4) ability to complete self-assessment questionnaires. In compliance with published guidelines (15), six patients were excluded and referred back to their general practitioners due to the presence of any of the following conditions: serious spinal disease (e.g., cancer or infection), neurologic deficit, unexplained weight loss, fever, structural deformity, or symptoms of systemic disease. All conditions were classified using ICD-11 codes.

To minimize treatment bias, a stepwise recruitment process was used. While the treating physician informed eligible patients (*n* = 354) about the study, consent forms and questionnaires were returned directly to the statistics office. The coordinator assigned identification numbers and managed data collection, ensuring the treating physician was blinded to participants' enrollment status. This procedural separation allowed clinical decisions to be made solely based on patients' symptoms and clinical needs, without any potential influence of study participation knowledge. This blinding process was crucial for obtaining unbiased results.

Acupuncture treatments were administered in a family medical practice setting by an experienced western-trained medical doctor specialized in both general practice and Traditional Chinese Medicine. The practitioner had experience as a family medicine physician since 1974 and as a pathologist since 1980, with formal acupuncture training including one year of full-time study at Shanghai University of Traditional Chinese Medicine (2008–2009) and Master Degree TCM specialization at Technical University Munich (2012–2016), practicing combined family medicine and acupuncture since 2008.

Acupuncture treatments followed established TCM principles. Treatments were individualized based on patient presentation but typically included a combination of local points at the pain site and distal points according to channel theory. Standardized points according to ICD-coded health conditions (Supplementary Table S1, S2) and practitioner-selected points were used bilaterally. Sterile, disposable stainless-steel needles (diameter 0.20 mm, length 40 mm, Green Nature, manufactured by Suzhou ZhongJing Life & Science Technology Ltd, Wujiang, China) were used.

Needles were inserted with the specific aim of achieving De-Qi sensation, which as previously described, indicates the activation of Qi flow in the meridians. Care was taken to elicit this sensation through brief manual manipulation of the needles after insertion, with varying depths depending on point location. De-Qi was confirmed when patients reported any of the characteristic sensations mentioned earlier (soreness, numbness, distension, heaviness, or warmth radiating from the needle site). These sensations were explained to patients before treatment, and verbal confirmation was obtained during needle manipulation. Sessions lasted 45–60 min, with the first visit including intake interview and thorough physical examination lasting 60–90 min. Session frequency was tailored to patient needs, with patients free to terminate treatment at any point.

Post-treatment health was evaluated against Norwegian national normal health standards. We examined the dose effect of acupuncture by assessing health changes across different treatment frequencies (28–30), to explore a potential dose-response relationship of acupuncture.

Given the high incidence of chronic pain in patients aged 65 and older, we also examined the differences in treatment outcomes across age groups.

Propensity score matching (PSM) mimicked randomization and reduced confounding bias. Logistic regression predicted propensity scores based on baseline characteristics across treatment frequencies. A 1:1 greedy matching algorithm balanced samples across treatment frequencies, as confirmed by standardized mean differences (SMD) < 0.1 and *p*-values > .05, indicating the absence of significant differences between matched groups.

**Figure 1:**
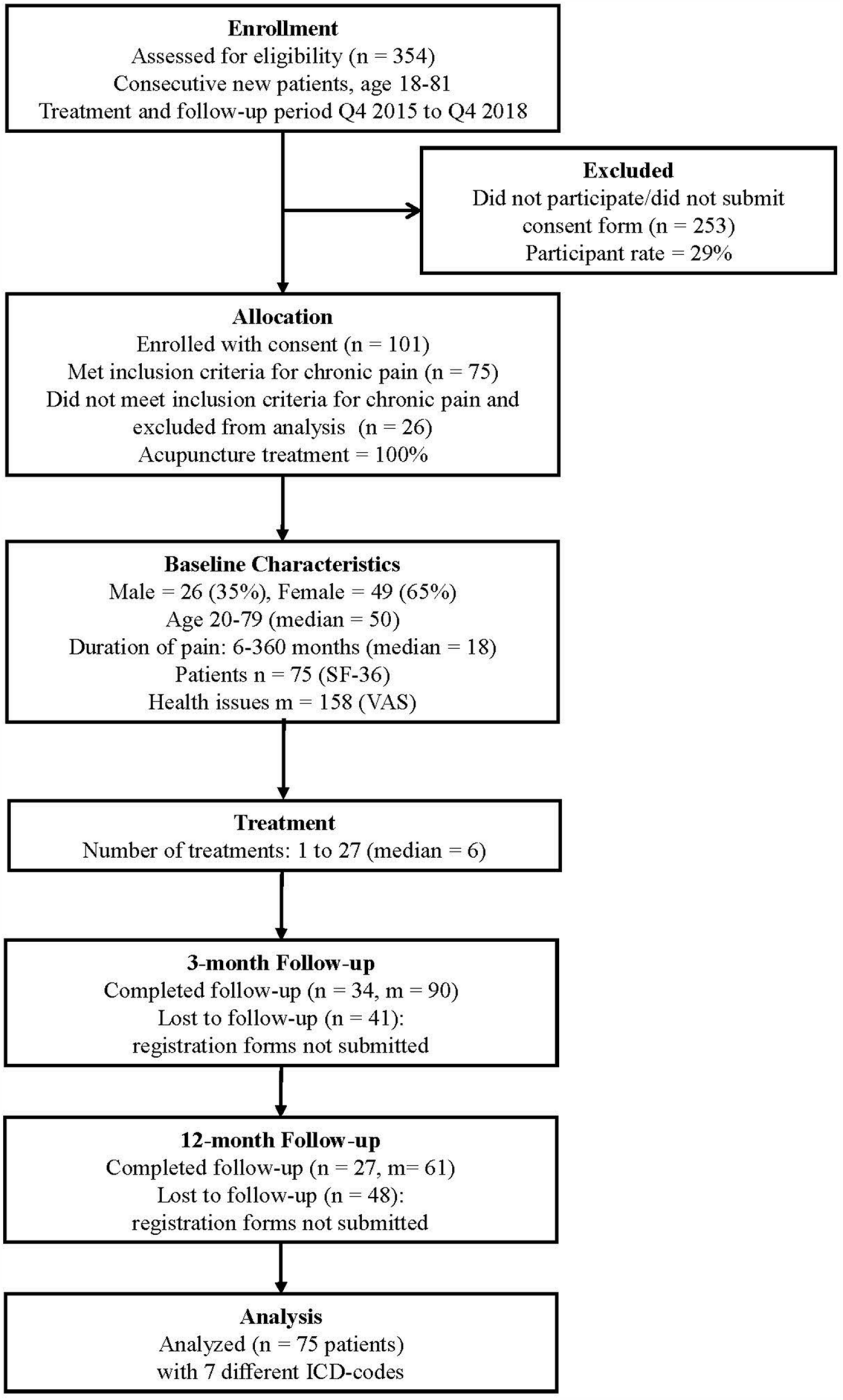
Study design and participant flow chart.

The enrollment and study design are outlined in Figure 1. Of the 354 patients, 101 (29% participation rate) provided written consent and submitted registration forms. This high participation rate aligns with the 2017 Norwegian reference values for the 36-item Short-Form Health Survey (31). Of these 101 consented participants, 75 met the specific inclusion criteria for chronic pain conditions affecting the neck, shoulder, back, hips, and knees, (Supplementary Table S1) and were included in the final analysis. The remaining 26 patients had conditions other than chronic pain in the specified anatomical regions.

Patients self-assessed pain using the Visual Analogue Scale (VAS) and health using the Short Form 36 Health Survey (SF-36), including Bodily Pain (BP), Physical Component Summary (PCS), and Mental Component Summary (MCS) before treatment, and at 3-month and 12-month follow-up periods. These were compared to evaluate improvement, Norwegian national health standards, and effect sizes.

Among the 75 included patients, 15 submitted only VAS forms but not SF-36 forms at baseline. The number of health issues assessed with VAS (m: count of health issues) and patients completing SF-36 (*n*: count of patients) at each time point were: pre-treatment (*m* = 144, *n* = 60), 3-month follow-up (*m* = 90, *n* = 34), and 12-month follow-up (*m* = 61, *n* = 27). This progressive decrease in response rates is typical in longitudinal studies with extended follow-up periods.

### Statistical analysis

SPSS Statistics v29 was used. Descriptive statistics were assessed for all features. Linear mixed-effects regression was used to evaluate changes in VAS and SF-36 scores over pre-treatment, 3-month, and 12-month intervals, and accommodated repeated measurements and multiple health issues. Treatment effect size was quantified using Cohen's d statistic.

Normal health status post-treatment was assessed by comparing PCS scores to Norway's national health data (32). The range for normal health in the general population was defined as ±3 T-scores from the population norm of 50. Thus, a PCS score ≥47 was set as the lower boundary of normal health, based on minimal clinically important differences and expert consultation (A. M. Garratt, Norwegian Institute of Public Health, personal communication, 2019).

Responders showed PCS improvements of ≥3 in the T-scores, while in strong responders the T-scores were ≥8 (Cohen's *d* ≥ 0.8), at the time of final observations (at 3 or 12 months). Patients who achieved complete cures showed PCS scores of ≥47, which reflected normal health. Health declines were defined as PCS decreases of ≥3 in T-scores. To assess the impact of initial health on treatment outcomes, patients were categorized by pre-treatment PCS scores as: very poor (<30), poor (30–39), and slightly poor health (40–47).

To investigate whether the observed association between acupuncture and improved health outcomes implied causation, Hill's criteria were applied (Supplementary Table S3) (25).

## Results

A total of 75 patients aged 20–79 (median 50) presented with 158 ICD-coded pain issues (median 2, range: 1–7); 65% of patients were women. The median pain duration was 18 months (range 6–360) and the median number of acupuncture treatments was 6 (range 1–27, mean 7.3, SD 4.6) (Table 1).

**Table 1:** Pre-Treatment characteristics of 75 patients in relation to Age and treatment frequency.

Features		All patients (*n* = 75)	Age 20–64 years (*n* = 63)	Age 65+ years (*n* = 12)	1–5 treatments (Full dataset, *n* = 17)	6+ treatments (Full dataset, *n* = 58)	1–5 treatments (PS matched dataset^†^, *n* = 7)	6+ treatments (PS matched dataset^†^, *n* = 7)
Sex								
Female		49 (65%)	44 (70%)	5 (42%)	11 (65%)	38 (66%)	6 (86%)	6 (86%)
Male		26 (35%)	19 (30%)	7 (58%)	6 (35%)	20 (34%)	1 (14%)	1 (14%)
Age in years	Range	20–79	20–64	65–79	20–68	20–79	20–65	32–78
	Median	50	43	73	44	52	42	56
	Mean	47.2	42.6	71.3	42.9	48.5	45.0	56.0
	SD	16.6	13.8	5.4	17.6	16.3	17.3	17.3
Duration of pain in months	Range	6–360	6–300	18–360	18–360	6–300	18–360	18–300
	Median	18	18	18	18	18	60	18
	Mean	42.9	40.4	56.0	85.8 ^‡^	30.4 ^‡^	103.7	58.3
	SD	70.4	63.7	101.2	100.8	53.6	123.0	106.6
Number of treatments	Range	1–27	1–27	3–17	1–5	6–27	1–5	6–19
	Median	6	6	6	3	6	4	6
	Mean	7.3	7.4	6.3	3.1	8.5	3.7	8.0
	SD	4.6	4.8	3.6	1.5	4.5	1.5	4.9
Number of health issues for all patients combined		158	133	25	34	124	15	16
Number of health issues per patient	Range	1–7	1–7	1–4	1–6	1–7	1–3	1–4
	Median	2	2	2	1	2	2	2
	Mean	2.1	2.1	2.1	2.0	2.1	2.1	2.3
	SD	1.3	1.3	1.0	1.4	1.3	0.9	1.3
Number of VAS forms pretreatment		144	122	22	31	113	15	16
Pain intensity VAS scores	Range	1–10	1–10	1–10	3–9	1–10	3–9	1–10
	Median	7	7	5.5	7	6	7	6
	Mean	6.2	6.2	5.8	6.4	6.1	6.2	6.4
	SD	2.5	2.5	2.6	1.7	2.7	2.1	2.7
Number of SF–36 forms pretreatment		60	53	7	14	46	7	7
Physical component summary	Range	12.3–57.5	12.3–57.5	36.8–49.7	12.3–55.1	14.6–57.5	12.3–55.1	24.3–49.7
	Median	37.9	35.5	39.1	35.8	38.3	38.0	42.4
	Mean	37.4	36.7	42.0	34.7	38.1	36.6	39.3
	SD	11.2	11.7	5.1	15.1	9.8	16.7	8.6
Mental component summary	Range	12.7–58.4	12.7–58.4	27.7–57.6	19.2–56.0	12.7–58.4	24.5–55.96	28.6–57.6
	Median	41.7	41.2	48.9	41.2	41.9	41.2	41.9
	Mean	40.6	39.9	46.4	40.5	40.7	41.7	43.1
	SD	11.0	11.0	9.8	11.2	11.0	12.1	10.4
Bodily pain	Range	15.0–59.8	15.0–59.8	28.1–52.5	19.6–57.3	15.0–59.8	19.6–57.3	25.0–59.8
	Median	42.2	41.9	45.7	41.3	43.1	42.5	45.6
	Mean	41.2	41.1	41.6	38.3	42.1	39.6	43.0
	SD	11.0	11.4	8.6	11.7	10.8	13.1	14.1

PS Matched dataset^†^: Pre-treatment characteristics were similar among age groups and between treatment frequency groups (1–5 vs. 6+ treatments), with the exception of a minor difference in pain duration (*p* = .03)^‡^ (Supplementary Table S4). Propensity score matching resolved this difference, ensuring that observed treatment effects were attributable to the treatment rather than to underlying differences between the groups.

Pre-treatment characteristics were similar across age groups and between treatment frequencies (1–5 vs. 6+ treatments), except for a minor difference in pain duration (*p* = .03). Propensity score matching resolved this difference (*p* = .34) (Supplementary Table S4).

Descriptive statistics for age groups and treatment frequency at baseline, and at 3-months and 12-months of follow-up are shown in Supplementary Table S5. No significant differences in VAS or PCS were observed among age groups (*p* = .33; *p* = .29), indicating that patients aged 65 and older also responded positively to acupuncture treatment. Therefore, further analyses included patients of all ages.

**Figure 2:**
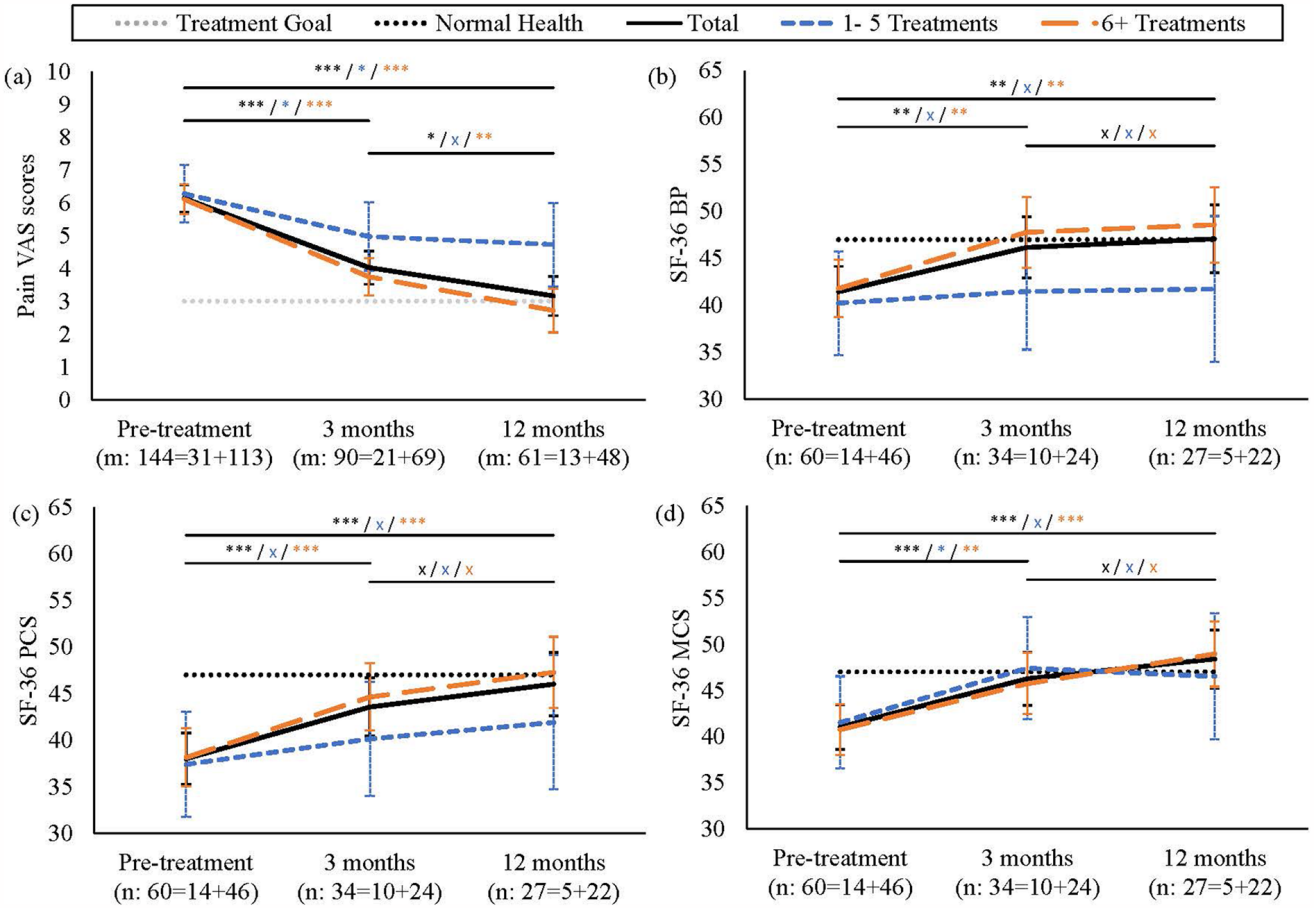
Pain intensity and SF-36 Trends Across Treatment Frequencies. **(a)** VAS and **(b–d)** SF-36 (BP, PCS, MCS) trends showing achievement of treatment goals (*p* < .001). Patients with 6+ treatments (orange long dashed line) showed substantial improvements at 12 months (*p* < .001) vs. lesser responses in the 1-5 treatments group (blue dashed line). Black solid line represents total patient averages. Dotted lines indicate normal health reference values and treatment goals. Significance levels: ***p < .001, **p < .01, *p < .05, x not significant. Error bars: 95% CI. Numbers below each time point show: m = total health issues assessed (VAS); *n* = total patients evaluated (SF-36). Values are further broken down into 1–5 treatments and 6+ treatments.

Linear mixed-effects regression analysis showed that sustained pain reduction was significant following treatment. VAS pain scores decreased from 6.15 at pre-treatment, to 4.02 at 3 months and 3.16 at 12 months (large effect size, *p* < .001), indicating that the effect was pronounced and sustained (Figure 2; Supplementary Table S6). Patients who received ≥6 treatments exhibited stronger responses, with scores decreasing by 3.39 points at 12 months (large effect size, *p* < .001). SF-36 BP, PCS and MCS also improved over the longer term (moderate-large effect sizes, *p* < .001) (Supplementary Table S7). For details on the statistical model, see Supplementary Table S6 and S7. Pre- and post-treatment PCS score comparisons (paired t-test, *p* < .001) showed large effect sizes (Cohen's *d* = 0.8) and high statistical power (0.99), confirming robust health improvements (Supplementary Table S7, Figure S1).

**Figure 3:**
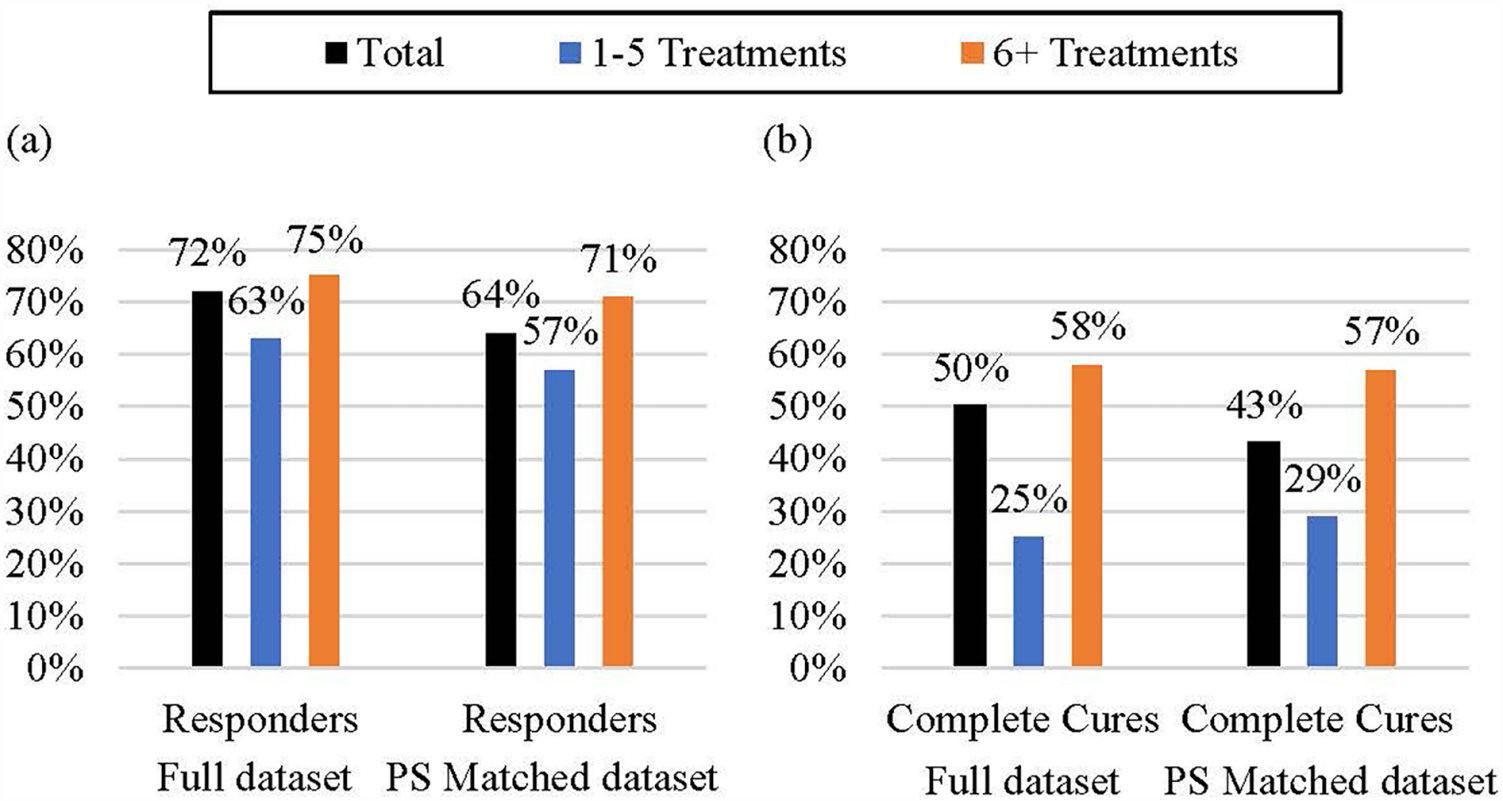
Response and complete cure rates according to treatment frequency. **(a)** Responders: Patients with significant physical health improvements post-treatment **(b)** Complete Cures: Patients who regained normal physical health are shown (PCS≥47). Results for full dataset and propensity score matched dataset compared 1-5 vs. 6+ treatments. Proportions of responses were similar in full and propensity-matched datasets (Chi-square test, *p* = .59), supporting the robustness of the findings. The 6+ treatment group consistently demonstrated higher response rates and substantially higher complete cure rates in both analytic approaches.

Post-treatment, 72% of participants were strong responders, according to SF-36 evaluations (≥8 T-score improvement), with 50% achieving complete cures (PCS≥47) and reaching normal physical health standards. The percentage of “improved outcomes” (72%) significantly exceeded “worsened outcomes” (3%) (*p* < .001). In total, 28% of participants were non-responders (Figure 3; Supplementary Table S8). Patients with ≥6 treatments showed higher response rates (75% strong responders, 58% complete cures; 17% achieving near-normal health; Supplementary Table S8); proportions of responses were similar in full and propensity-matched datasets (*p* = .59; Supplementary Table S8), supporting the robustness of the findings.

Responder proportions were similar across initial physical health status (very poor, PCS<30), poor (30–39), and slightly decreased (40–47); *p* = .34), indicating an overall positive treatment effect. However, fewer patients with poor or very poor initial health achieved complete cures compared with those with slightly decreased health (*p* = .004).

Across this analysis, associations between acupuncture and improved health outcomes, using the Hill criteria (Supplementary Table S3), were consistent with causation over association. A visual overview of the study population, design, and key findings is presented in Supplementary Data Sheet 1.

## Discussion

This prospective study of the effect of De-Qi acupuncture treatment administered by an experienced medical practitioner/TCM specialist was conducted in a family medicine practice in patients with long-lasting chronic pain (6–360 months, median 18 months), who were resistant to conventional therapies. The findings indicated that, at 12-months of follow-up, most patients were almost completely cured and reached normal health levels comparable to the healthy population. Furthermore, the therapeutic effect sizes were statistically large and strongly relevant from a clinical perspective. Importantly, patients receiving ≥6 treatments experienced greater benefits than those with 1–5 treatments and notable improvements were observed across all initial health statuses. Most improvement was achieved in the first three months of treatment (35% decrease in VAS pain scores), with an additional 14% pain reduction over the subsequent nine months.

In terms of the consequences of these findings for society, this study highlights the possibility of potentially alleviating the suffering of patients, while delivering cost savings for the national healthcare system. The effectiveness of acupuncture in patients aged >65 years is of particular clinical relevance. Comparisons of outcomes with Norwegian national health norms provided an objective assessment of health improvements from long-term follow-up and enhanced the robustness of the findings. Additionally, the dose-dependent effect of treatment frequency, including substantial improvements with ≥6 treatments, is important for the motivation of future patients to undertake at least 6 treatments.

Meta-analyses have found acupuncture to be superior to no-acupuncture controls for pain conditions (33–35). Comparisons with sham acupuncture controls have yielded mixed results. Effect sizes in previous studies (0.42–0.57 compared to no-acupuncture controls) are comparable to, though slightly lower than those observed in the current study. The larger effect sizes in our study might reflect the emphasis on De-Qi sensation, which some researchers suggest may enhance therapeutic outcomes (16). Importantly, the observation that Hill criteria suggest causation over association is consistent with these collective findings.

Our study aligns with and expands upon previous research on acupuncture for the treatment of chronic pain in a primary care setting. In 2013, McKee et al. reported significant pain and physical health improvements post-acupuncture (36). Even though our participants had experienced pain of longer duration (6 vs. 3 months), pain reductions were more pronounced (6.2–3.2 at 12 months vs. 6.8–5.5 at 6 months) and more patients were responders (72%–75% vs. 40%–50%). These improved outcomes may stem from differences in treatment protocols: we emphasized “De-Qi”, or “Qi-arrival” at acupuncture and employed an experienced practitioner, while McKee et al. employed students under supervision. Moreover, the “cure” outcomes in the present study were achieved with fewer treatments (mean 7.3 vs. 9.7), thus supporting the assertion that acupuncture treatment quality is crucial. While we did not measure medication use, reduced pain levels indicate a potential for decreased reliance on pharmacological treatments, which could lead to a reduction in associated side effects. Together, these findings indicate that De-Qi acupuncture by experienced practitioners following traditional methods provides greater benefits for chronic pain in primary care than previously reported.

The working mechanisms of pain reduction by acupuncture have been extensively studied (37) and include endorphins release stimulation*,* the way pain signals are processed in the spinal cord, have local effects at the site of needle insertion and systemic effects throughout the body, enhance immune function and activation of brain areas associated with pain processing. Recent studies have summarized cytokines and pain (38). Modulation of pro-inflammatory cytokine signaling between immune, glial, and neural cells is integral to the development of pain (39). Improvement in pain relief and function in mild to moderate knee osteoarthritis is partly mediated by changes of major inflammatory factors TNF-α, IL-1β and IL-13 (40). In rats, the underlying mechanism of acupuncture treatment of migraine is either the regulation of hyperalgesia and neurotransmitters, or the reduction of inflammatory factors (41). On the other hand, Kinin-kallikrein and renin-angiotensin systems, through B1, B2 and AT2 receptors, potentiates paclitaxel-associated acute pain syndrome (P-APS) in mice. Antagonists for AT2R are potential alternatives to prevent P-APS (42). For a detailed explanation of the biological and neuroimmune mechanisms of acupuncture, see Supplementary Data Sheet 2.

Whether the skepticism toward TCM and acupuncture might be altered by findings such as those in the present study is unclear. However, the 2015 Nobel Prize in Physiology or Medicine was awarded to *Professor Youyou Tu* and has enhanced the acceptance of traditional medicine (43). Furthermore, in 2018, the World Health Organization included TM in its ICD-11 system. Currently, in Norway, non-medical health providers, such as midwives and physiotherapists, frequently use acupuncture and moxibustion. Pressure from these large occupational groups could promote the wider acceptance of acupuncture.

The cost to patients for 6 acupuncture treatments was about 450 USD in this study. With an almost complete recovery rate at one-year of follow-up (75%), the cost-benefit ratio appears favorable, though a formal cost-effectiveness analysis was beyond the scope of this study. In future, multicenter studies should independently investigate the effectiveness of acupuncture in isolation, and in combination with other therapeutic modalities, to develop comprehensive pain management strategies.

## Conclusions

Patients with long-term chronic pain who are resistant to conventional treatments often face unfavorable prognoses. In this observational real-world study, we found that routine acupuncture with De-Qi, in a family medicine practice, was associated with clinically significant improvements in 75% of patients, with many achieving normal health standards according to Norwegian population norms. Patients who received ≥6 treatments exhibited significantly better improvements than those receiving fewer treatments, indicating a dose-dependency effect. Importantly, patients >65 years responded very well, and even those with very poor initial health (PCS<40) showed improvements, although they did not reach the Norwegian normal health standard threshold.

Propensity score analysis helped control for known confounders, strengthening these associations. The Hill criteria analysis (25) supports a potential causal relationship between acupuncture and health improvements. However, we acknowledge the inherent limitations of our observational study design. The necessity of achieving De-Qi during acupuncture makes constructing a true control group challenging, as patients know when they feel this distinct sensation. Without a randomized control group, we cannot definitively rule out the influence of natural history, regression to the mean, or non-specific effects of treatment.

Nevertheless, given the chronic nature and treatment-resistant history of our patients' conditions (median duration 18 months), the substantial improvements observed warrant further investigation. Future multicenter studies should investigate the effectiveness of De-Qi acupuncture alone, as well as in combination with other therapeutic modalities, to develop comprehensive pain management strategies.

## Data Availability

The raw data supporting the conclusions of this article will be made available by the first author, without undue reservation.

## Supplementary material

Supplementary Data Sheet 1 Summary of study design, population characteristics, and main findings, including visual data highlights.

Supplementary Data Sheet 2 Illustrated overview of acupuncture's local, systemic, and neuroimmune effects, supported by current research citations.

Supplementary Material Supplementary Tables S1–S8, detailing ICD codes, acupuncture points, Hill's criteria for causation assessment, statistical analyses, longitudinal outcome data, and responder analyses. STRICTA 2010 acupuncture reporting guidelines checklist. STROBE statement checklist for observational studies.
