# Supplementary material for: The majority of Norwegian patients with treatment-resistant chronic pain regained normal national health standards within 12 months after De-Qi acupuncture - a prospective observational propensity score matched study
Source: Front Pain Res (Lausanne). 2025 Apr 8;6:1521466. doi: 10.3389/fpain.2025.1521466 (PMC12011868; doi:10.3389/fpain.2025.1521466)
Supplement: Supplementary Data Sheet 1 — Summary of study design, population characteristics, and main findings, including visual data highlights. [file Datasheet1.pdf]

The majority of Norwegian patients with treatment-resistant chronic pain regained normal national health standards within 12 months after de-qi acupuncture - A prospective observational propensity score matched study

METHOD

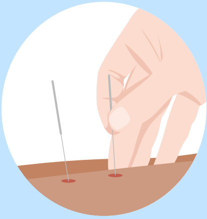

Qi-arrival Acupuncture for chronic pain (2015-2018)

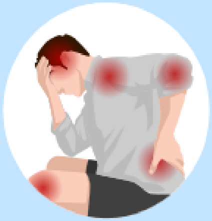

Pain (VAS) and health (SF-36) at baseline, 3, and 12 months

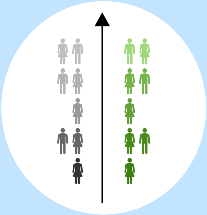

Propensity Score Matching: balanced patient characteristics between groups

RESULTS

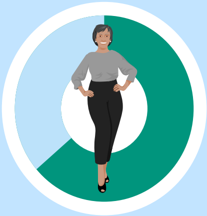

354 Patients
Median age 50
65% female
Median pain duration 18 months

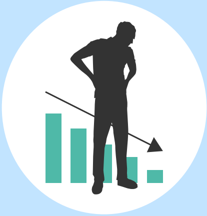

Pain (VAS):
6.2→4.0 (3mo)→3.2(12mo)
$p<0.001$, large effect size

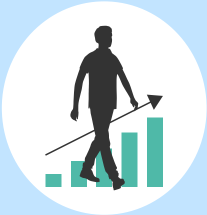

Health (SF-36):
58% complete cure
+17% near-normal health

KEY TAKE-AWAYS

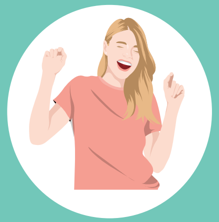

Sustained pain relief for pain unresponsive to conventional treatment

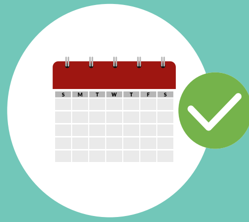

75% achieved cure or Near normal health, including patients aged 65+

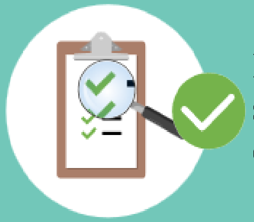

Hill criteria analysis supports acupuncture's causal effect

No financial support was received for the research, authorship, and/or publication of this article.

Authors:Lindberg Veronika, Baak Jan P.A. MD, PhD, FICP, FRCPath, FIAChon, DrHC(Antwerp), Professor of Pathology, Stavanger University Hospital, Norway

Frontiers in Pain Research
Non-Pharmacological Treatment of Pain
