# Supplementary material for: The majority of Norwegian patients with treatment-resistant chronic pain regained normal national health standards within 12 months after De-Qi acupuncture - a prospective observational propensity score matched study
Source: Front Pain Res (Lausanne). 2025 Apr 8;6:1521466. doi: 10.3389/fpain.2025.1521466 (PMC12011868; doi:10.3389/fpain.2025.1521466)
Supplement: Supplementary Data Sheet 2 — Illustrated overview of acupuncture's local, systemic, and neuroimmune effects, supported by current research citations. [file Datasheet2.pdf]

LOCAL AND SYSTEMIC MECHANISMS OF PAIN REDUCTION BY ACUPUNCTURE

Insights into Neuroimmune Modulation and Inflammatory Pathways

Studied in rodents and human

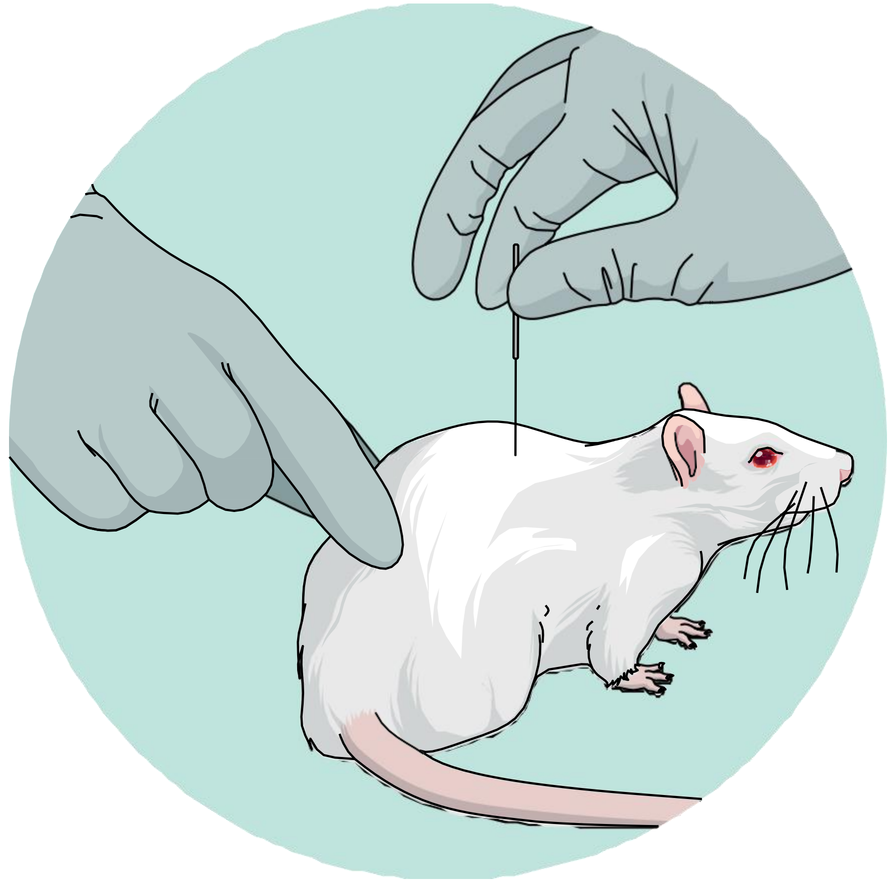

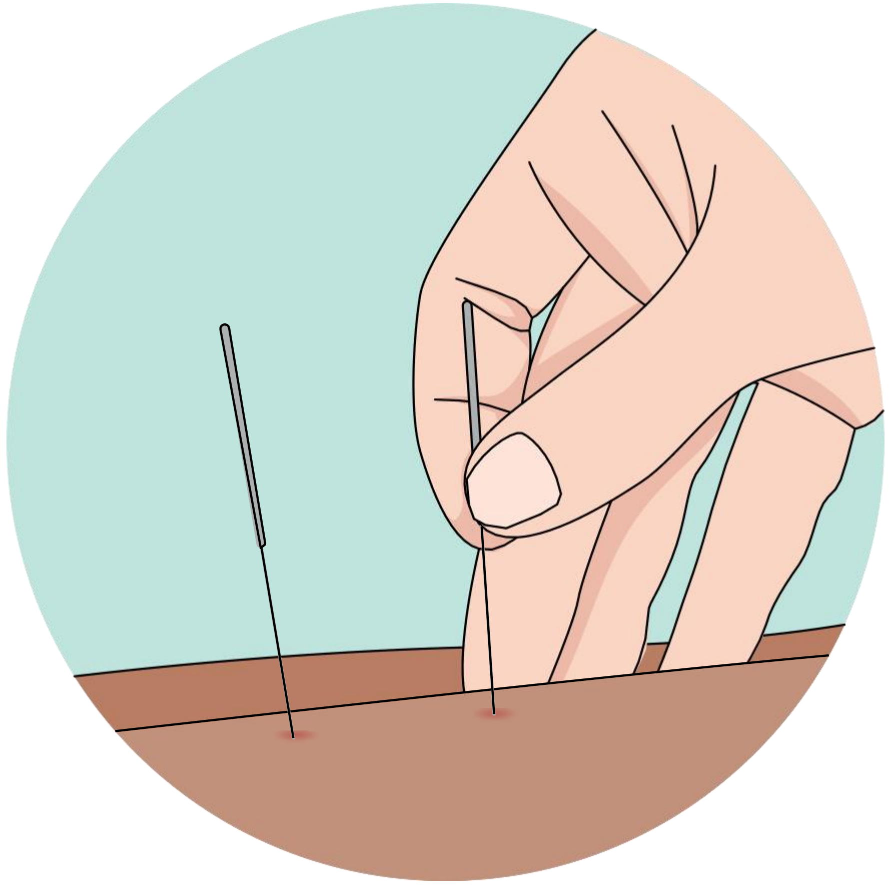

Understanding Acupuncture's Role in Pain Management

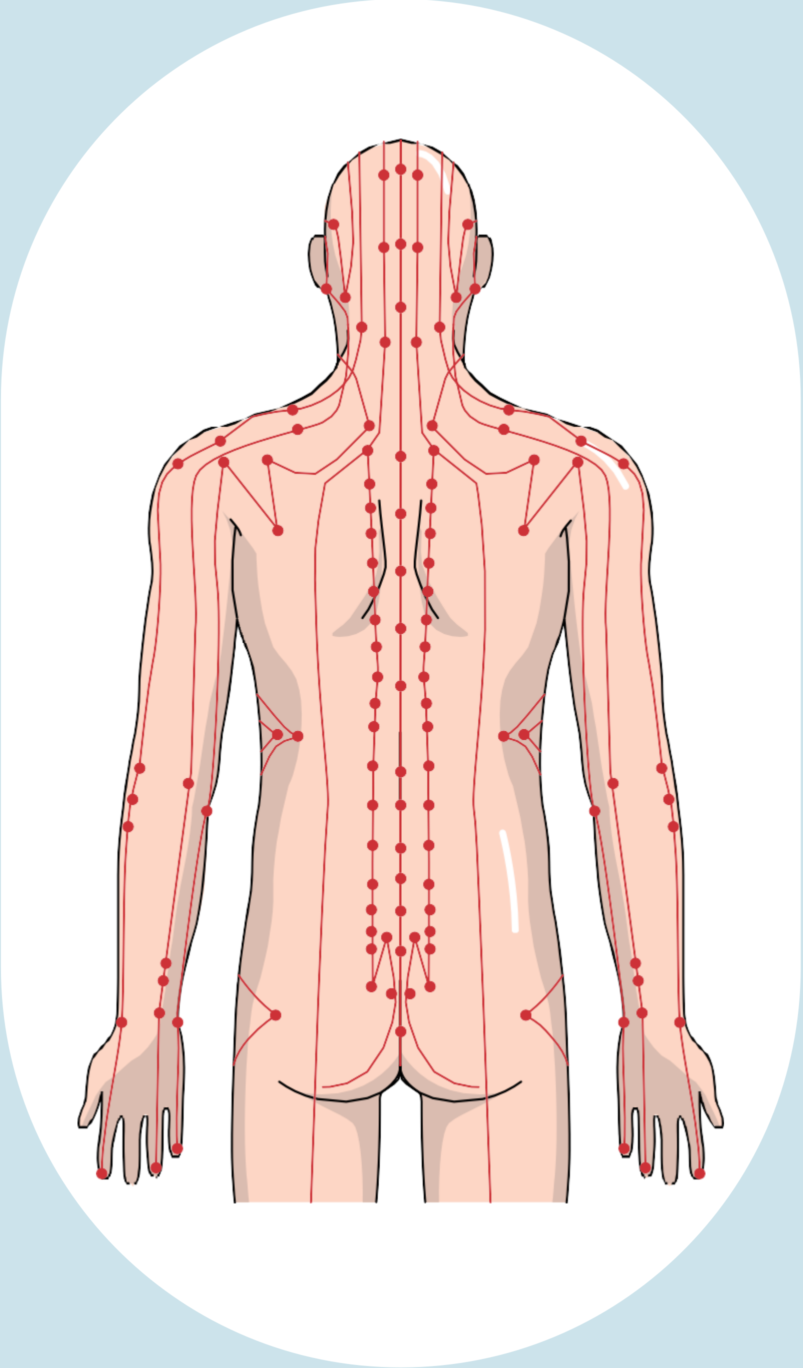

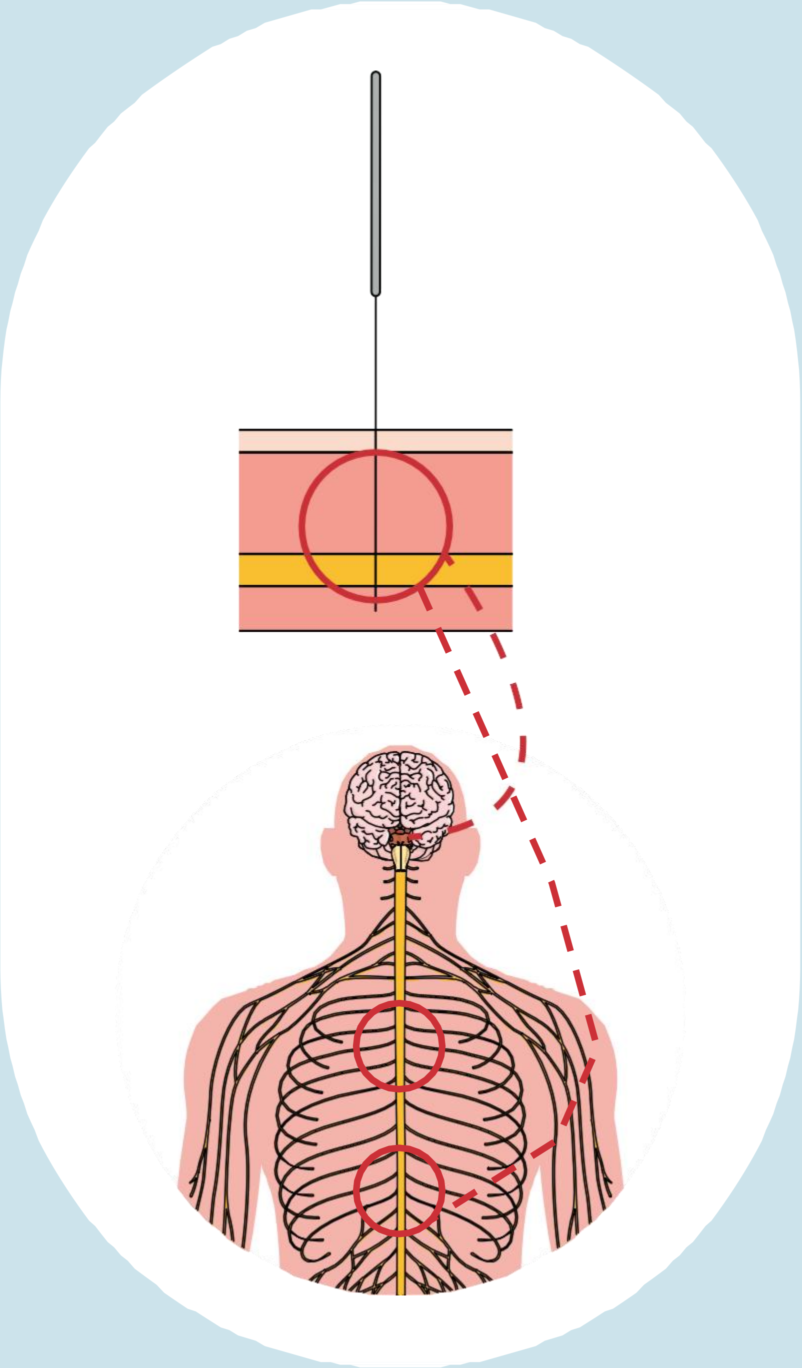

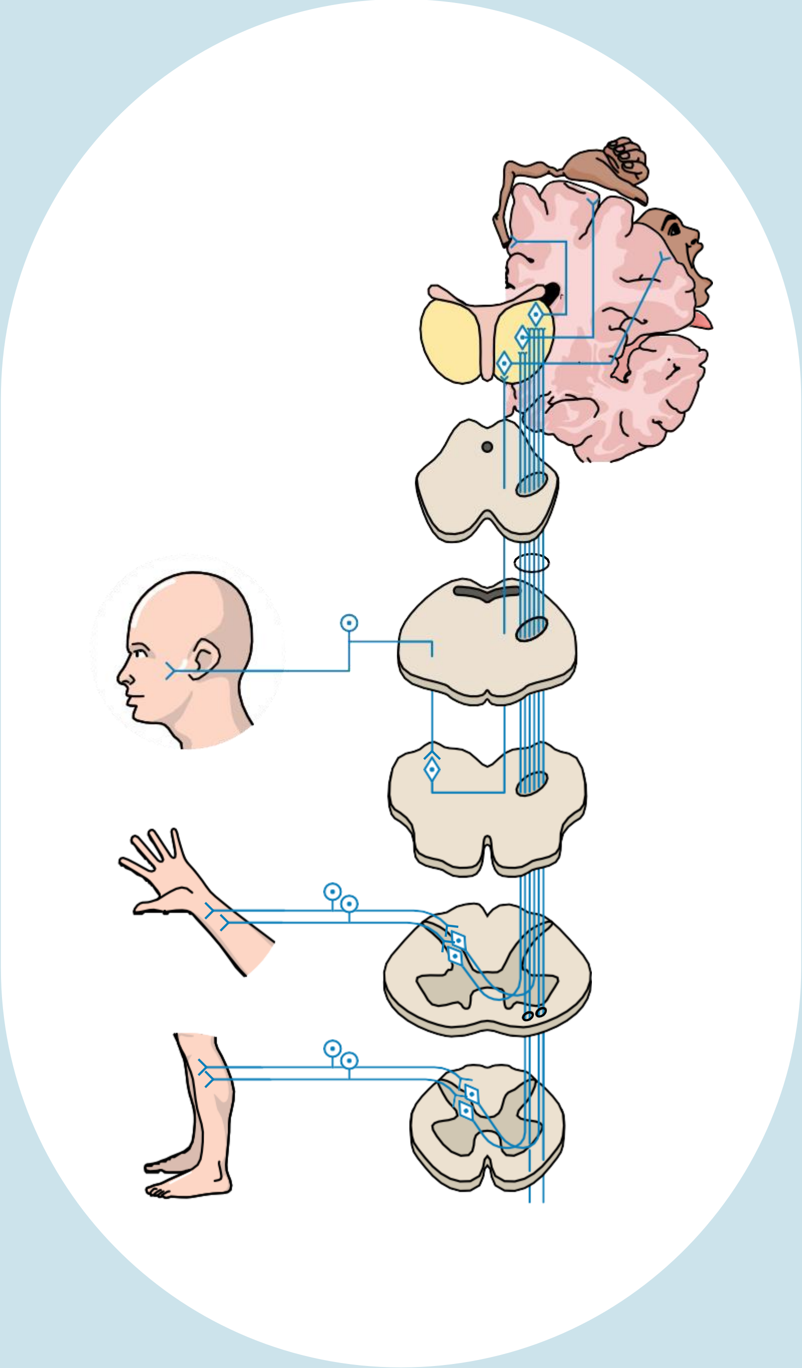

- Acupuncture is increasingly recognized as a therapeutic approach for pain management.
- Extensive research confirms its local and systemic effects in reducing pain, validated through multiple pathways such as spinal cord modulation, endorphin release, and local tissue responses.
- Multi-pathway mechanisms involve the intersection of neurological, immunological, and inflammatory processes.
- Clinical applications demonstrate evidence-based success in treating chronic pain, migraines, osteoarthritis, and lower back pain.

Bridge between traditional practice and modern scientific understanding

Vanderwall AG et al., 2019; Pinto LG et al., 2021; Smith et al., 2024.

Immediate and Local Tissue Responses to Needle Insertion

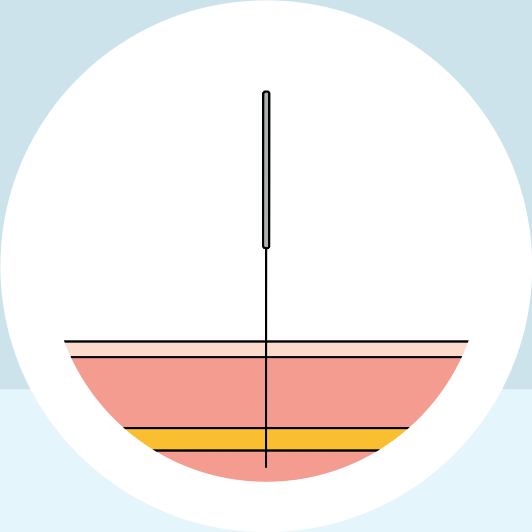

Microtrauma Response

- Initial microtrauma triggers localized healing cascade
- Activation of local immune cells and nociceptors
- Release of inflammatory mediators (substance P, bradykinin)

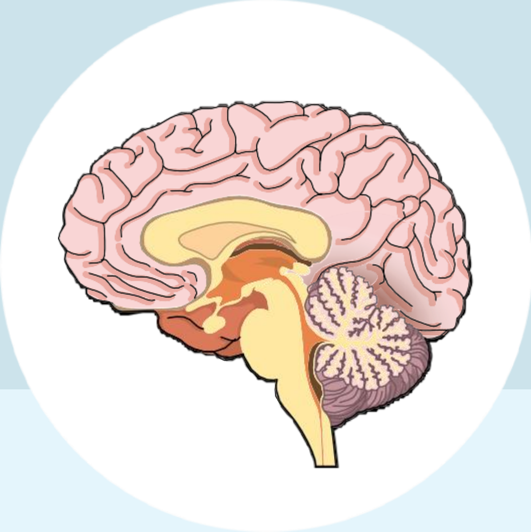

Vascular Changes

- Enhanced microcirculation and blood flow
- Release of vasodilatory substances
- Improved tissue oxygenation and nutrient delivery

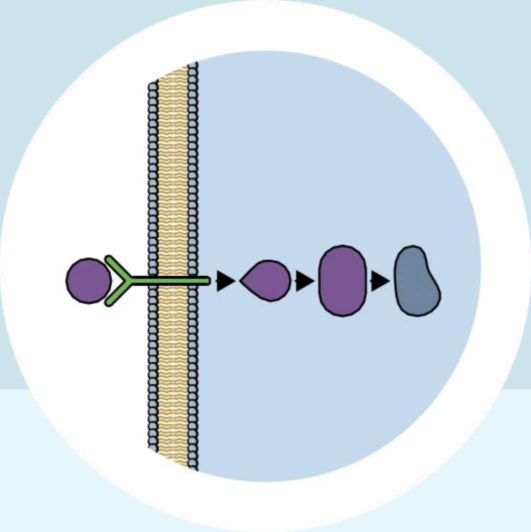

Molecular Signaling

- Local endorphin and enkephalin release
- Modulation of inflammatory cytokines
- Activation of mechanoreceptors and A-delta fibers

Vanderwall AG et al., 2019

Mechanisms of Spinal Cord Pain Processing: Gate Control, Synaptic Plasticity, and Signal Integration

- **Gate Control Mechanism Activation:** Pain signal modulation at the dorsal horn.
- **Signal Transmission Modulation:** Alteration of ascending pain pathways.
- **Inhibitory Interneurons:** Activation and regulation of pain signal suppression.
- **Synaptic Plasticity:** Changes in synaptic strength within pain pathways.
- **Signal Integration:** Coordination of ascending nociceptive and descending inhibitory signals.

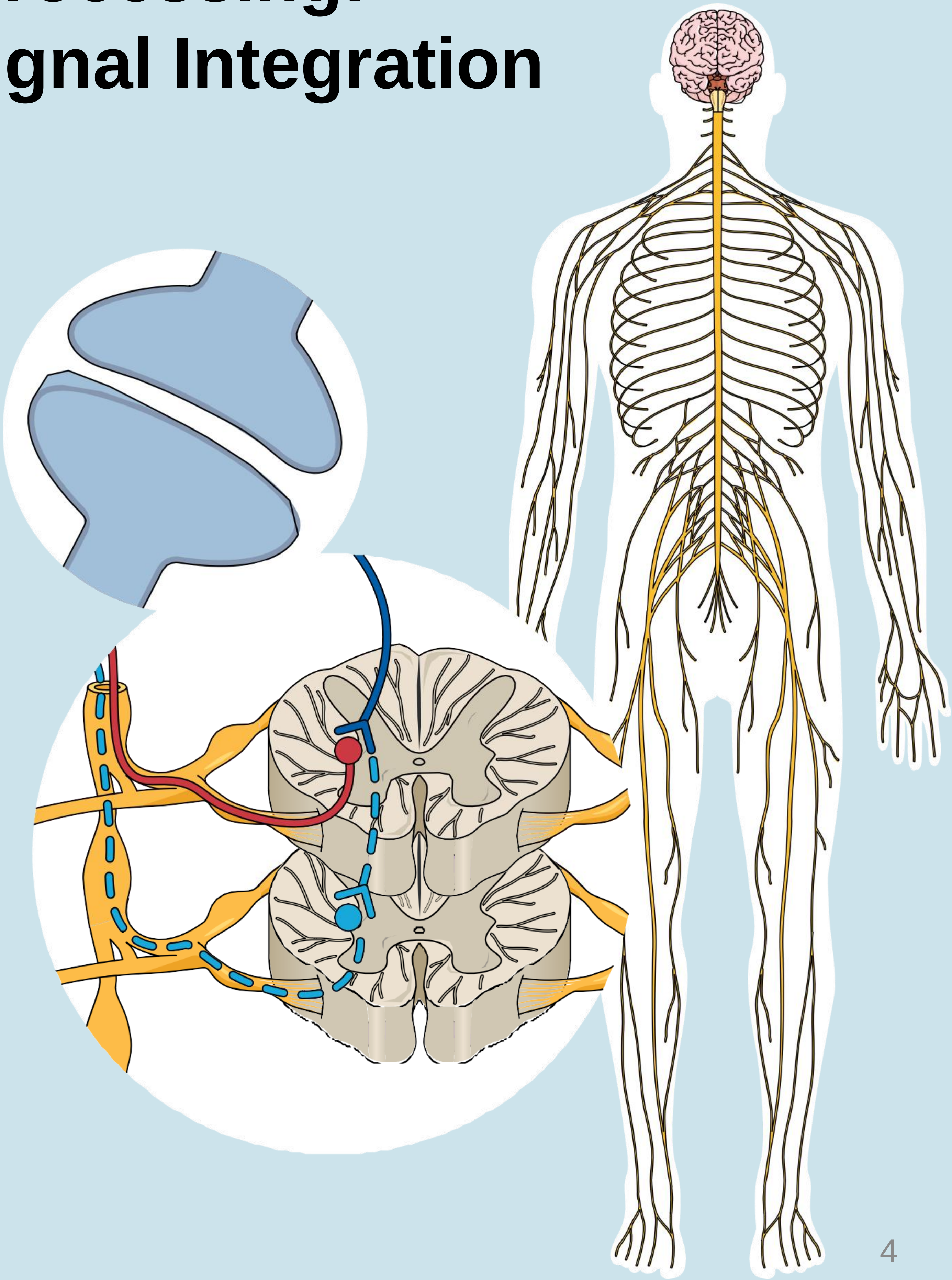

Vanderwall AG et al., 2019

Neural and Immune System Responses to Acupuncture

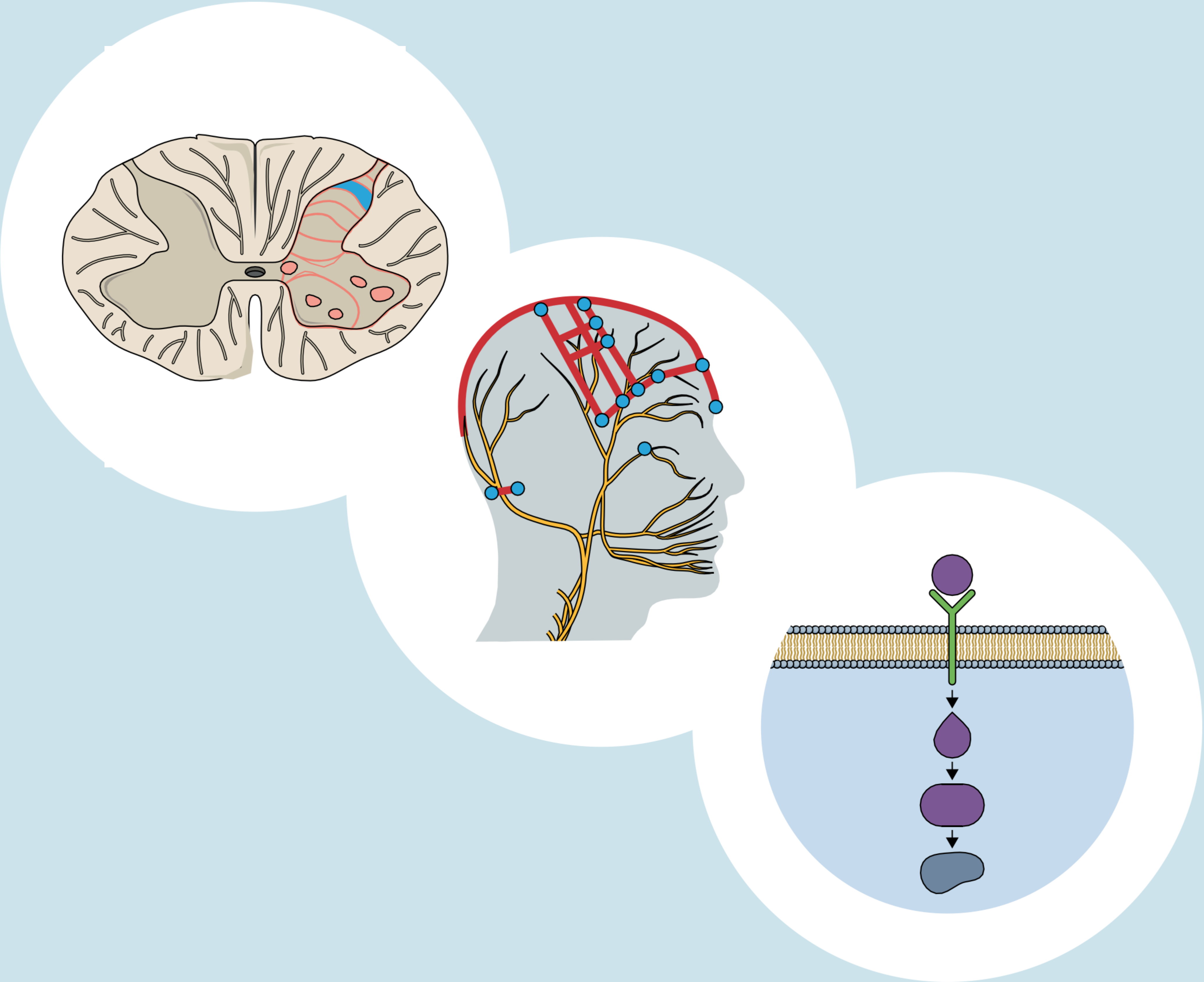

Neural Modulation

- Spinal cord gate control mechanism activation
- Descending pain inhibitory pathway stimulation
- Dorsal horn neuroplasticity changes
- Engagement of pain matrix regions (anterior cingulate, insula)
- Modulation of limbic system activity

Neuroimmune Integration

- Hypothalamic-pituitary-adrenal axis activation
- Enhanced systemic anti-inflammatory responses
- Increased regulatory T-cell activity

Vanderwall AG et al., 2019; Pinto LG et al., 2021; Shi GX et al., 2020.

Cytokine Networks in Pain Modulation

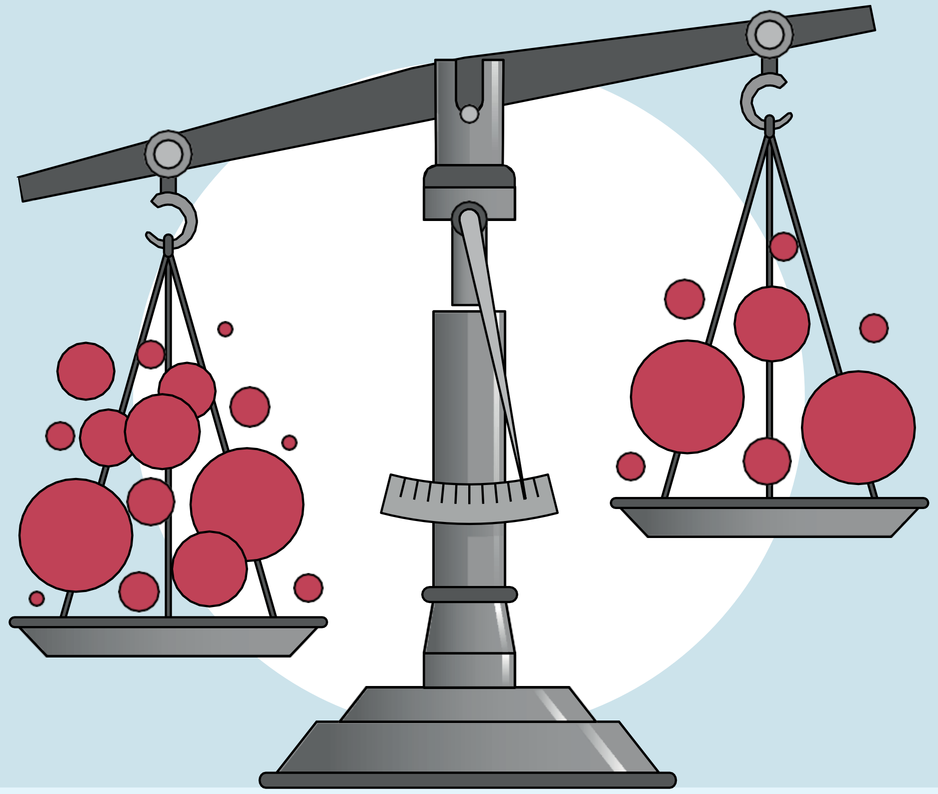

Pro-inflammatory Cascade

- TNF-α as primary initiator of inflammatory response
- IL-1β amplification of pain signaling
- IL-13 contribution to chronic pain states

Cellular Interactions

- Microglial activation and sensitization
- Astrocyte-neuron signaling pathways
- T-cell mediated inflammatory responses

Clinical Relevance

- Demonstrated effects in osteoarthritis
- Temporal changes in cytokine profiles
- Correlation with pain reduction outcomes

Vanderwall AG et al., 2019; Pinto LG et al., 2021; Shi GX et al., 2020.

Acupuncture's Multi-Modal Effects in Migraine Treatment

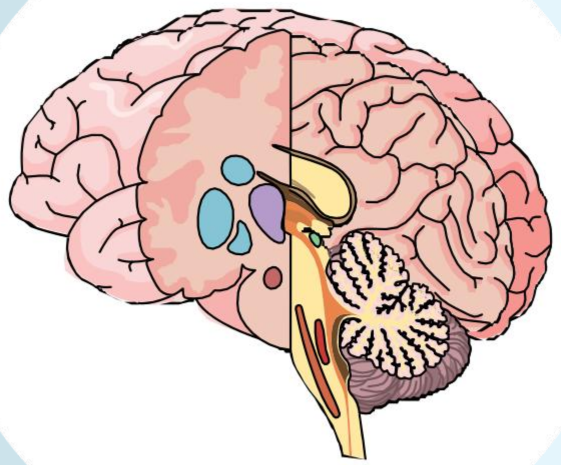

Vascular Mechanisms

- Calcitonin gene-related peptide (CGRP) modulation
- Cerebral vessel tone regulation
- Blood-brain barrier permeability changes

Neurological Effects

- Cortical spreading depression inhibition
- Trigeminal nerve activation reduction
- Pain threshold modulation in trigeminal nuclei

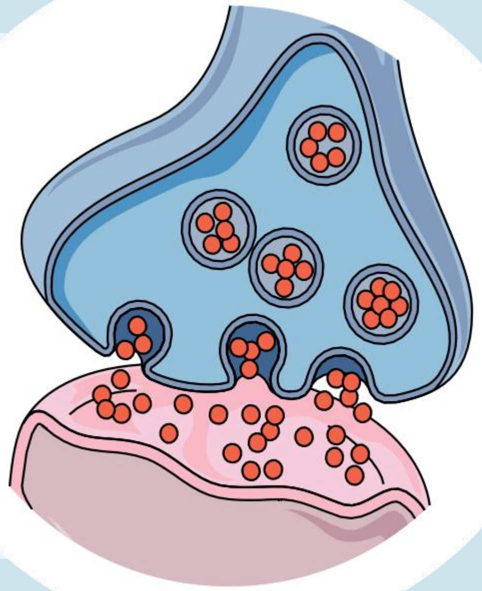

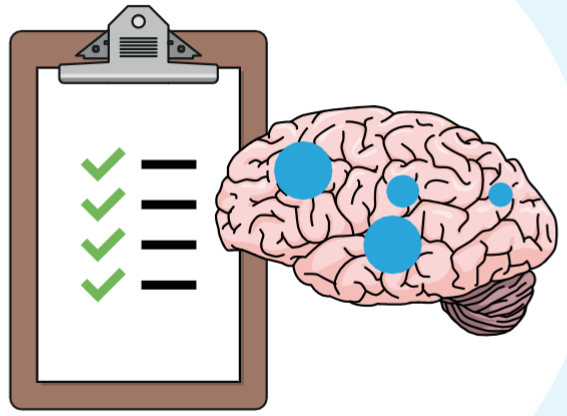

Biochemical Modulation

- Serotonin level regulation
- Substance P reduction
- Glutamate/GABA balance restoration

Sun S et al., 2023.

CLINICAL APPLICATION - OSTEOARTHRITIS

Mechanisms in Osteoarthritis Pain Management

Acupuncture points

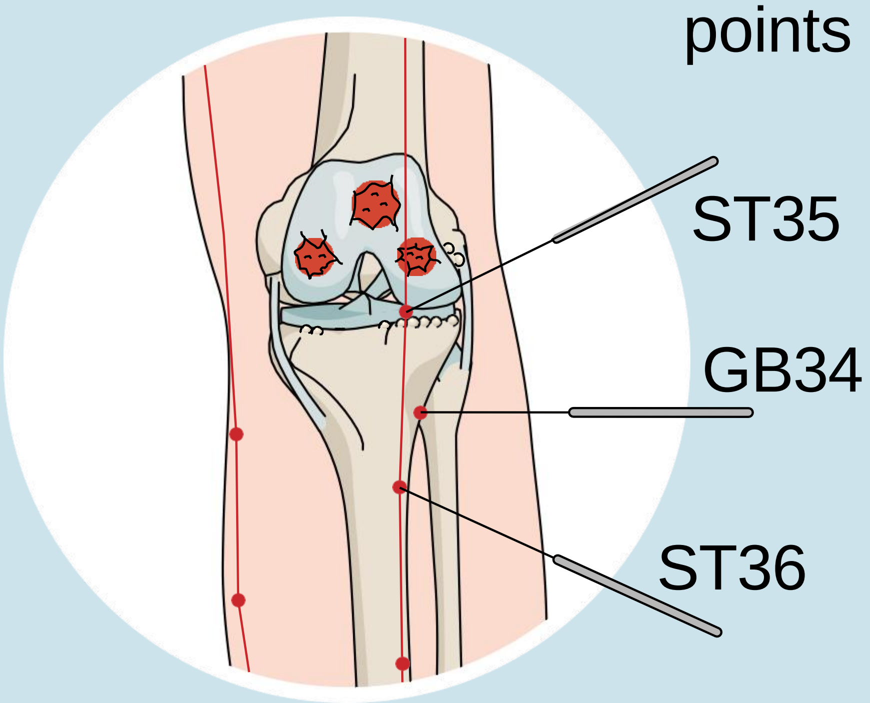

ST35
GB34
ST36

Sensory neurons

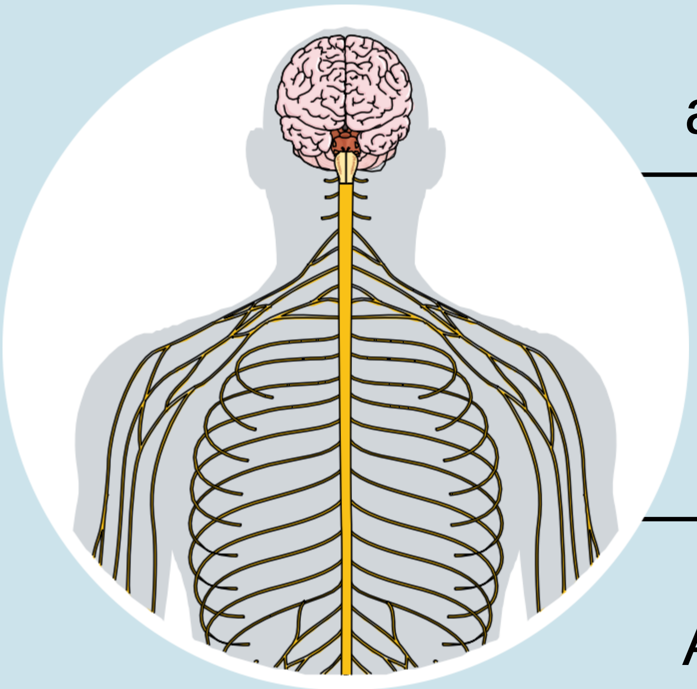

EA Electro acupuncture

MA Acupuncture

Inflammatory Markers

TNF-α ↓ (EA>MA)

IL-13 ↑ (EA=MA)

IL-1β ↓ (EA=MA)

Clinical Outcomes (8 weeks)

**VAS Pain Reduction**	EA: 26.8 units, MA: 25.4 units
**WOMAC Function Improvement**	EA: 9.5 points, MA: 8.3 points

Cartilage Degradation

MMP-3 ↓ (EA=MA)

MMP-13 ↓ (EA=MA)

·Demonstrated improvement in pain and function

·Modulation of specific inflammatory markers:
- TNF-α reduction
- IL-1β regulation
- IL-13 pathway effects

·Clinical outcome correlations

Shi GX, Tu JF, Wang TQ, Yang JW, Wang LQ, Lin LL, Wang Y, Li YT, Liu CZ. Effect of Electro-Acupuncture (EA) and Manual Acupuncture (MA) on Markers of Inflammation in Knee Osteoarthritis. J Pain Res. 2020 Aug 26;13:2171-2179.

KININ-KALLIKREIN SYSTEM AND PAIN MODULATION IN ACUPUNCTURE

Interplay Between Acupuncture and Pain-Related Pathways

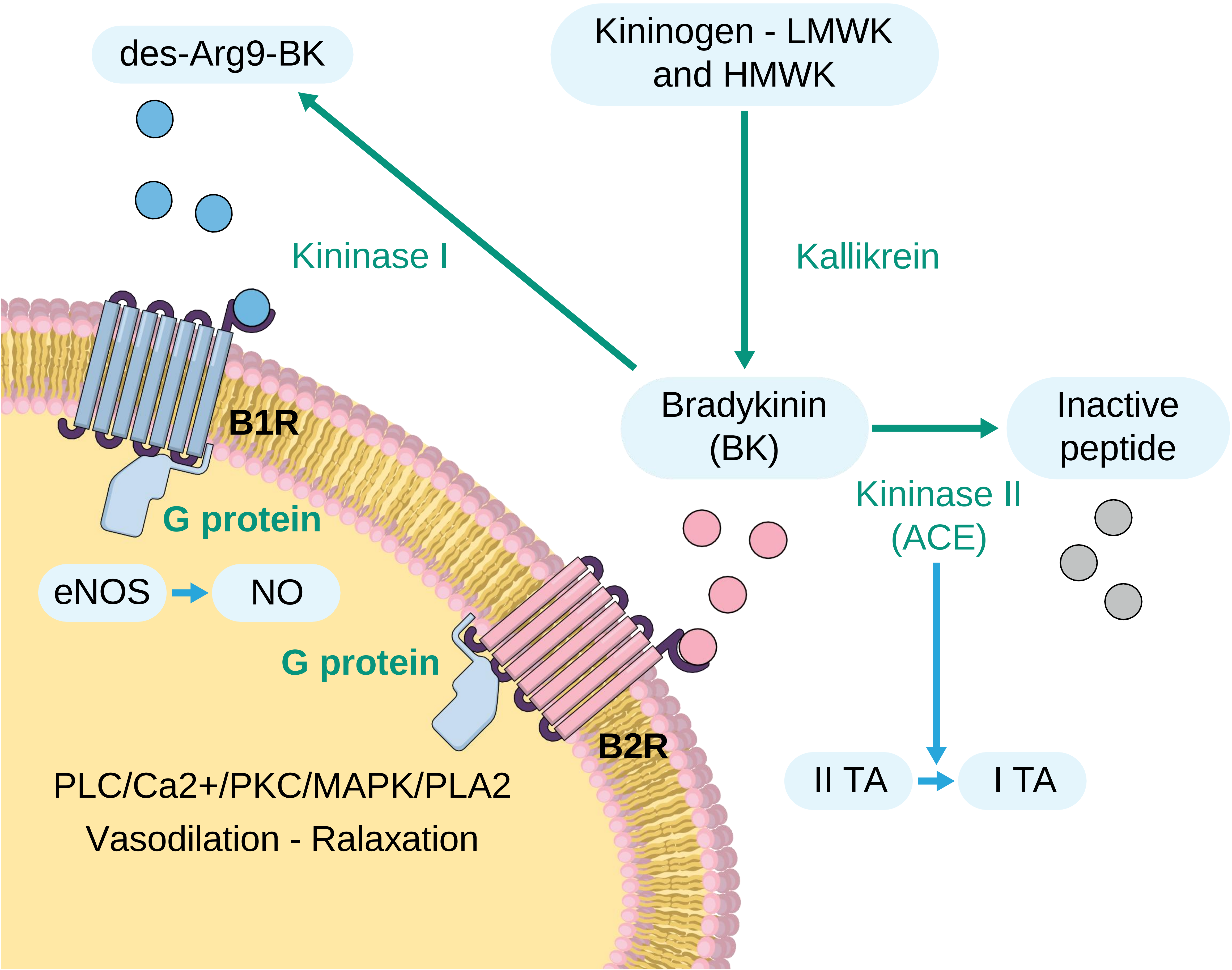

- Kinin-Kallikrein System: Key role in inflammation and pain signaling through B1 and B2 receptor activation.
- Renin-Angiotensin System (RAS): Implications in pain modulation, with AT2 receptors showing potential antinociceptive effects.
- Chemotherapy-Induced Pain: Kinin-kallikrein and RAS exacerbate paclitaxel-associated acute pain syndrome (P-APS). AT2 receptor antagonists emerge as potential therapies for preventing P-APS.
- Acupuncture Effects: Reduction in inflammatory mediators, modulation of neurotransmitter systems, and central regulation of pain processing pathways align with these mechanisms.
- Neuropathic Pain Relief: EA decreases TRPV1 expression, alleviating pain in dorsal root ganglia and spinal cord regions.

REFERENCES BY RESEARCH FOCUS

A. FOUNDATIONAL ACUPUNCTURE RESEARCH
1. State of Contemporary Acupuncture:
- **Smith CL, et al. (2024)**
  - **Focus: Current state of acupuncture in the United States**
  - **Journal: J Pain Res. 2024;17:3329-3354**
  - **Key contribution: Overview of acupuncture mechanisms and clinical validation**

B. PAIN & CYTOKINE MECHANISMS
1. Cytokine Signaling Overview:
- **Pinto LG, et al. (2021)**
  - **Focus: Editorial on cytokines and pain**
  - **Journal: Front Immunol. 2021;12:788578**
  - **Key contribution: Framework for understanding cytokine roles in pain**

2. Anti-inflammatory Signaling:
- **Vanderwall AG, Milligan ED. (2019)**
  - **Focus: Endogenous anti-inflammatory signaling in pain management**
  - **Journal: Front Immunol. 2019;10:3009**
  - **Key contribution: Cytokine signaling between immune, glial, and neural cells**

C. CLINICAL APPLICATIONS
1. Osteoarthritis Treatment:
- **Shi GX, et al. (2020)**
  - **Focus: Electro-acupuncture and manual acupuncture effects**
  - **Journal: J Pain Res. 2020;13:2171-2179**
  - **Key contribution: Changes in TNF-α, IL-1β, IL-13 in knee osteoarthritis**

2. Migraine Management:
- **Sun S, et al. (2023)**
  - **Focus: Analgesic effects in migraine rats**
  - **Journal: J Pain Res. 2023;16:2525-2542**

Key contribution: Hyperalgesia regulation and inflammatory factor reduction

D. RELATED PAIN RESEARCH
1. Chemotherapy-induced Pain:
- **Zanata GC, et al. (2021)**
  - **Focus: Paclitaxel-associated acute pain syndrome**
  - **Journal: Eur J Pain. 2021;25(1):189-198**
  - **Key contribution: Role of bradykinin and AT2 receptors in P-APS**
  - **Note: Not directly related to acupuncture but provides insight into receptor-mediated pain mechanisms**

Pinto LG, Pinho-Ribeiro FA, Verri WA Jr. Editorial: Cytokines and Pain. Front Immunol. 2021 Oct 21;12:788578. doi: 10.3389/fimmu.2021.788578. PMID: 34745153; PMCID: PMC8566714

Vanderwall AG, Milligan ED. Cytokines in Pain: Harnessing Endogenous Anti-Inflammatory Signaling for Improved Pain Management. Front Immunol. 2019 Dec 23;10:3009. doi: 10.3389/fimmu.2019.03009. PMID: 31921220; PMCID: PMC6935995

Shi GX, Tu JF, Wang TQ, Yang JW, Wang LQ, Lin LL, Wang Y, Li YT, Liu CZ. Effect of Electro-Acupuncture (EA) and Manual Acupuncture (MA) on Markers of Inflammation in Knee Osteoarthritis. J Pain Res. 2020 Aug 26;13:2171-2179. doi: 10.2147/JPR.S256950. PMID: 32904642; PMCID: PMC7457556

Sun S, Liu L, Zhou M, Liu Y, Sun M, Zhao L. The Analgesic Effect and Potential Mechanisms of Acupuncture for Migraine Rats: A Systematic Review and Meta-Analysis. J Pain Res. 2023 Jul 24;16:2525-2542. doi: 10.2147/JPR.S422050. PMID: 37521010; PMCID: PMC10378646

Smith CL, Reddy B, Wolf CM, Schnyer RN, St John K, Conboy L, Stone J, Lao L. The State of 21st Century Acupuncture in the United States. J Pain Res. 2024 Oct 10;17:3329-3354. doi: 10.2147/JPR.S469491. PMID: 39403098; PMCID: PMC11472758.

Zanata GC, Pinto LG, da Silva NR, Lopes AHP, de Oliveira FFB, Schivo IRS, Cunha FQ, McNaughton P, Cunha TM, Silva RL. Blockade of bradykinin receptors or angiotensin II type 2 receptor prevents paclitaxel-associated acute pain syndrome in mice. Eur J Pain. 2021 Jan;25(1):189-198. doi: 10.1002/ejp.1660. Epub 2020 Oct 6. PMID: 32965065
