# Supplementary material for: The majority of Norwegian patients with treatment-resistant chronic pain regained normal national health standards within 12 months after De-Qi acupuncture - a prospective observational propensity score matched study
Source: Front Pain Res (Lausanne). 2025 Apr 8;6:1521466. doi: 10.3389/fpain.2025.1521466 (PMC12011868; doi:10.3389/fpain.2025.1521466)
Supplement: Supplementary Material — Supplementary Tables S1–S8, detailing ICD codes, acupuncture points, Hill's criteria for causation assessment, statistical analyses, longitudinal outcome data, and responder analyses. STRICTA 2010 acupuncture reporting guidelines checklist. STROBE statement checklist for observational studies. [file Table1.docx]

**Table S1** *Health Issues of Study Participants According to ICD Codes.*

| ICD Code | ICD Description | Number of Patients |
| --- | --- | --- |
| M25.51 | Shoulder Pain | 7 |
| M25.55 | Pain in Hip | 10 |
| M25.56 | Pain in Knee | 15 |
| M54.2 | Cervicalgia | 9 |
| M54.5 | Lower Back Pain | 68 |
| M54.6 | Pain in Thoracic Spine | 24 |
| M54.9 | Dorsalgia, Unspecified | 10 |
| Total | Distinct Patients | 75 |

**Note:** "Number of Patients" indicates the count of patients diagnosed with each condition.
The total distinct count of patients accounts for individuals diagnosed with one or more conditions.

**Table S2** *Acupuncture Points Used for Different Pain Conditions by Anatomical Location and ICD Code*

| Location | Western name | Chinese name |
| --- | --- | --- |
| Shoulder Pain (M25.51) | LI15 | Jianyu |
|  | SJ14 | Sianliao |
|  | SI9 | Jianzhen |
|  | SI10 | Naoshu |
|  | LI4 | Hegu |
|  | TB5 | Waiguan |
|  | GB34 | Yanglingquan |
|  | ST38 | Tiaokou |
| Pain in Hip (M25.55) | GB30 | Huantiao |
|  | GB29 | Zhonglushu |
|  | BL54 | Zhibian |
|  | BL36 | Chengfu |
|  | ST31 | Biguan |
|  | GB34 | Yanglingquan |
|  | BL40 | Weizhong |
|  | KI3 | Taixi |
|  | SP6 | Sanyinjiao |
| Pain in Knee (M25.56) | ST35 | Dubi |
|  | Extra point knee eye | Xiyan |
|  | SP9 | Yinlingquan |
|  | GB34 | Yanglingquan |
|  | BL40 | Weizhong |
|  | ST36 | Zusanli |
|  | KI3 | Taixi |
|  | LR3 | Taichong |
|  | BL60 | Kulun |
| Cervicalgia (M54.2) | GB20 | Fengchi |
|  | BL10 | Tianzhu |
|  | SI15 | Jianzhongshu |
|  | GV14 | Dazhui |
|  | LI4 | Hegu |
|  | TB5 | Waiguan |
|  | SI3 | Houxi |
|  | GB39 | Xuanzhong |
| Lower Back Pain (M54.5) | BL23 | Shenshu |
|  | BL25 | Dachangshu |
|  | BL40 | Weizhong |
|  | GV3 | Yaoyanguan |
|  | GV4 | Mingmen |
| Lower Back Pain (M54.5) - Sacro-iliacal joint | BL27 | Xiaoxiangshu |
|  | BL28 | Pangguangshu |
|  | BL29 | Zhonglushu |
|  | BL30 | Beihuanshu |
|  | GB39 | Huantiao |
|  | CV3 | Yaoyangguan |
|  | BL40 | Weizhong |
|  | BL60 | Kulun |
|  | GB34 | Yanglingquan |
|  | KI3 | Taixi |
| Pain in Thoracic Spine (M54.6) | BL23 | Shenshu |
|  | BL25 | Dachangshu |
|  | BL40 | Weizhong |
|  | GV3 | Yaoyanguan |
|  | GV4 | Mingmen |
| Dorsalgia, Unspecified (M54.9) | BL23 | Shenshu |
|  | BL25 | Dachangshu |
|  | BL40 | Weizhong |
|  | GV3 | Yaoyanguan |
|  | GV4 | Mingmen |

**Table S3** *Suggesting Causation Between Acupuncture and Improved Health Outcomes Using Hill's Criteria.*

| Hill's Criterion | Description |
| --- | --- |
| Strength | The large effect sizes observed in our study (Cohen's d:0.80-0.91 for physical health improvements and 0.80- 1.35 for pain reduction) demonstrate clinically significant improvements in quality of life and pain reduction, providing strong evidence of a robust association. |
| Consistency | Our results are consistent with those from other research demonstrating similar improvements across different populations and settings. |
| Specificity | The specific improvements seen in patients receiving acupuncture suggest a direct effect of the treatment. |
| Temporality | The timing of improvements, which occurred within three months and lasted for 12 months, supports a causal relationship. |
| Biological Gradient | Patients receiving 6+ treatments showed significantly greater improvements (75% responders, 58% complete cures) compared to those receiving only 1-5 treatments (63% responders, 25% complete cures), demonstrating a clear dose-response relationship. |
| Plausibility | The known mechanisms of acupuncture, such as pain pathway modulation and opioid release, make such results plausible. See Supplementary File “Graphical ACUPUNCTURE MECHANISMS.pdf.” |
| Coherence | The findings align with existing knowledge about acupuncture’s effects on chronic pain. |
| Experiment | Although our study is observational, the use of propensity score matching, and robust statistical analyses enhances experimental rigor and minimizes biases. The increased proportions of patients cured with more treatments support causation. |
| Analogy | The analogy to similar treatments, such as transcutaneous electrical nerve stimulation (TENS), which also modulates pain pathways and provides relief for chronic pain, further supports our conclusions. |

**Note:** The strong association between acupuncture treatment and improved outcomes suggests a causal relationship according to Hill’s criteria[23]. The evidence supports this conclusion through the criteria of strength, consistency, specificity, temporality, biological gradient, plausibility, coherence, experiment, and analogy, collectively making a compelling case for the effectiveness of acupuncture in treating chronic pain.

The application of Hill's criteria within the GRADE framework[24] strengthens our confidence in the causal relationship between acupuncture and observed health improvements. While observational studies typically rank below RCTs, GRADE acknowledges that strong associations, dose-response relationships, and consistency with existing knowledge can upgrade evidence quality from observational studies. As emphasized in the referred study above, GRADE specifically allows for upgrading the quality of evidence from observational studies when they demonstrate particularly strong associations, as observed in our findings with large effect sizes for both pain reduction and physical health improvements.

**Table S4** *Logistic Regression Analysis of Pre-Treatment Characteristics Across Age and Treatment Frequency Groups.*

| Stratification Group | Feature | Logistic Regression Coefficients | Standard Error | Wald Test Statistic | DF | p-value | | Odds Ratio |
| --- | --- | --- | --- | --- | --- | --- | --- | --- |
| Age Groups^†^: 20–64 vs. 65+ | Female | -1.14 | 0.94 | 1.45 | 1 | .23 | x | 0.32 |
|  | Pain duration in Months | 0.01 | 0.01 | 1.56 | 1 | .21 | x | 1.01 |
|  | Number of Treatments | -0.17 | 0.20 | 0.68 | 1 | .41 | x | 0.85 |
|  | VAS | 0.07 | 0.25 | 0.09 | 1 | .77 | x | 1.08 |
|  | Physical Component Summary | 0.07 | 0.09 | 0.57 | 1 | .45 | x | 1.07 |
|  | Mental Component Summary | 0.03 | 0.07 | 0.15 | 1 | .70 | x | 1.03 |
|  | Bodily Pain | -0.03 | 0.09 | 0.13 | 1 | .72 | x | 0.97 |
|  | Constant | -3.65 | 4.21 | 0.75 | 1 | .39 | x | 0.03 |
| Number of Treatments^‡^:  1–5 vs. 6+  (Full Dataset) | Female | -0.49 | 0.77 | 0.40 | 1 | .53 | x | 0.62 |
|  | Age 65+ | -1.05 | 1.02 | 1.06 | 1 | .30 | x | 0.35 |
|  | Pain duration in Months | -0.01 | 0.01 | 4.91 | 1 | .03^§^ | * | 0.99 |
|  | VAS | -0.23 | 0.19 | 1.45 | 1 | .23 | x | 0.80 |
|  | Physical Component Summary | 0.07 | 0.06 | 1.11 | 1 | .29 | x | 1.07 |
|  | Mental Component Summary | -0.02 | 0.05 | 0.20 | 1 | .66 | x | 0.98 |
|  | Bodily Pain | -0.03 | 0.06 | 0.30 | 1 | .58 | x | 0.97 |
|  | Constant | 3.25 | 2.64 | 1.51 | 1 | .28 | x | 25.67 |
| Number of Treatments:  1–5 vs. 6+  (Propensity Score Matched Dataset^¶^) | Female | 1.14 | 2.60 | 0.19 | 1 | .66 | x | 3.13 |
|  | Age 65+ | 1.15 | 2.37 | 0.24 | 1 | .63 | x | 3.16 |
|  | Pain duration in Months | -0.01 | 0.01 | 0.91 | 1 | .34 | x | 0.99 |
|  | VAS | 0.14 | 0.43 | 0.10 | 1 | .75 | x | 1.15 |
|  | Physical Component Summary | -0.02 | 0.13 | 0.04 | 1 | .85 | x | 0.98 |
|  | Mental Component Summary | 0.01 | 0.10 | 0.00 | 1 | .95 | x | 1.01 |
|  | Bodily Pain | 0.06 | 0.12 | 0.26 | 1 | .61 | x | 1.06 |
|  | Constant | -3.53 | 6.49 | 0.30 | 1 | .59 | x | 0.03 |

**Age Groups**^†^: Pre–treatment features do not differ significantly between the two age groups (Chi-squared test χ² = 7.87, DF = 7, p = .34), indicating good balance.
**Number of Treatments**^‡^: Overall, pre-treatment characteristics show no significant differences between the treatment frequency groups (Chi-squared test χ² = 10.94, DF = 7, p = .14); however, there was a small but significant difference in **pain duration**^§^ between the treatment frequency groups (1–5 treatments vs. 6+ treatments, Wald test *Z* = 4.91, DF = 1, p = .03).
**Propensity Score Matched Dataset**^¶^: Pain duration differences were resolved after matching (Wald test *Z* = 0.91, DF = 1, p = .34), ensuring observed treatment effects are likely due to the treatment itself rather than underlying differences between the groups. Baseline characteristics are balanced for all features in the propensity score matched dataset (Chi-squared test χ² = 1.36, DF = 7, p = .99).
**Significance Levels:** "x" denotes no statistically significant difference at p < .05, * indicates statistically significant differences at p < .05.

**Table S5**

*Descriptive Summary of VAS and SF-36 Pain Scores and Physical and Mental Summary Scores Across Time by Age and Treatment Frequency.*

| Measure | Time | All Health Issues | | |  | Age 20–64 Years | | | Age 65+ Years | | |  | 1–5 Treatments | | | 6+ Treatments | | |
| --- | --- | --- | --- | --- | --- | --- | --- | --- | --- | --- | --- | --- | --- | --- | --- | --- | --- | --- |
|  |  | m | Mean | SD |  | m | Mean | SD | m | Mean | SD |  | m | Mean | SD | m | Mean | SD |
| Pain intensity VAS scores  (Full Dataset) | Pre-treatment | 144 | 6.15 | 2.51 |  | 122 | 6.20 | 2.50 | 22 | 5.82 | 2.59 |  | 31 | 6.42 | 1.71 | 113 | 6.07 | 2.69 |
|  | 3-month | 90 | 4.21 | 2.52 |  | 75 | 4.13 | 2.52 | 15 | 4.60 | 2.59 |  | 21 | 4.95 | 2.31 | 69 | 3.99 | 2.56 |
|  | 12-month | 61 | 3.48 | 2.62 |  | 56 | 3.57 | 2.56 | 5 | 2.40 | 3.36 |  | 13 | 5.23 | 2.35 | 48 | 3.00 | 2.51 |
| Pain intensity VAS scores  (PS Matched Dataset) | Pre-treatment | 31 | 6.29 | 2.38 |  | n.a.^†^ | n.a. | n.a. | n.a. | n.a. | n.a. |  | 15 | 6.20 | 2.08 | 16 | 6.37 | 2.70 |
|  | 3-month | 30 | 4.50 | 2.45 |  | n.a. | n.a. | n.a. | n.a. | n.a. | n.a. |  | 14 | 5.36 | 1.86 | 16 | 3.75 | 2.70 |
|  | 12-month | 14 | 4.57 | 3.01 |  | n.a. | n.a. | n.a. | n.a. | n.a. | n.a. |  | 9 | 6.44 | 1.51 | 5 | 1.20 | 1.64 |
|  | Time | All Patients | | |  | Age 20–64 Years | | | Age 65+ Years | | |  | 1–5 Treatments | | | 6+ Treatments | | |
|  |  | n | Mean | SD |  | n | Mean | SD | n | Mean | SD |  | n | Mean | SD | n | Mean | SD |
| SF-36 BP Scores  (Full Dataset) | Pre-treatment | 60 | 41.22 | 11.00 |  | 53 | 41.17 | 11.35 | 7 | 41.57 | 8.59 |  | 14 | 38.33 | 11.66 | 46 | 42.10 | 10.77 |
|  | 3-month | 34 | 45.82 | 11.31 |  | 28 | 46.32 | 11.99 | 6 | 43.47 | 7.76 |  | 10 | 40.63 | 14.00 | 24 | 47.98 | 9.50 |
|  | 12-month | 27 | 46.52 | 11.40 |  | 24 | 46.79 | 11.17 | 3 | 44.34 | 15.69 |  | 5 | 39.18 | 12.34 | 22 | 48.19 | 10.78 |
| SF-36 MCS Scores  (Full Dataset) | Pre-treatment | 60 | 40.62 | 10.95 |  | 53 | 39.85 | 10.94 | 7 | 46.41 | 9.78 |  | 14 | 40.52 | 11.22 | 46 | 40.65 | 10.99 |
|  | 3-month | 34 | 45.73 | 8.17 |  | 28 | 45.13 | 8.68 | 6 | 48.54 | 4.76 |  | 10 | 47.41 | 10.24 | 24 | 45.04 | 7.29 |
|  | 12-month | 27 | 48.44 | 8.85 |  | 24 | 47.88 | 8.82 | 3 | 52.90 | 9.52 |  | 5 | 45.79 | 11.35 | 22 | 49.04 | 8.39 |
| SF-36 PCS Scores  (Full Dataset) | Pre-treatment | 60 | 37.35 | 11.19 |  | 53 | 36.73 | 11.65 | 7 | 41.99 | 5.10 |  | 14 | 34.74 | 15.08 | 46 | 38.14 | 9.79 |
|  | 3-month | 34 | 43.15 | 11.57 |  | 28 | 42.53 | 12.27 | 6 | 46.08 | 7.57 |  | 10 | 39.35 | 17.16 | 24 | 44.74 | 8.23 |
|  | 12-month | 27 | 45.64 | 12.16 |  | 24 | 45.16 | 12.08 | 3 | 49.48 | 14.78 |  | 5 | 38.49 | 15.38 | 22 | 47.27 | 11.09 |
| SF-36 PCS Scores | Pre-treatment | 14 | 37.91 | 12.82 |  | n.a. | n.a. | n.a. | n.a. | n.a. | n.a. |  | 7 | 36.56 | 16.67 | 7 | 39.27 | 8.60 |
| (PS Matched Dataset) | 3-month | 13 | 40.41 | 12.89 |  | n.a. | n.a. | n.a. | n.a. | n.a. | n.a. |  | 6 | 35.33 | 14.91 | 7 | 44.76 | 9.94 |
|  | 12-month | 7 | 42.82 | 13.79 |  | n.a. | n.a. | n.a. | n.a. | n.a. | n.a. |  | 4 | 42.02 | 15.22 | 3 | 43.87 | 14.84 |

**Counts**: ‘m’ represents the count of observed health issues, and ‘n’ is the number of patients. Propensity score-matched samples between the 1-5 and the 6+ treatment group are presented for VAS and SF-36 PCS. Significant changes in pain scores (VAS and BP) are detailed in Table S6, while significant changes in SF-36 Summary scores (PCS and MCS) are detailed in Table S7.
**n.a.** ^†^: Pre-treatment features did not differ between age groups, eliminating the need for propensity score matching. Furthermore, neither VAS nor PCS trends differed significantly between the two age groups (Linear Mixed-Effect Regression, VAS trends: F = 0.33, DF = 198, p = .57; PCS trends: F = 1.14, DF = 77, p = 0.29). Therefore, age differences are not evaluated further.
**Model Details:** In the linear mixed-effects regression model, means are adjusted for multiple observations from the same patient. The fixed effects are the intercept, time, number of treatments (1-5, 6+), age (20-64, 65+), and their interaction with time. Random effects are patient-specific intercepts, accounting for individual baseline variability. The model uses maximum likelihood estimation, Satterthwaite approximation for degrees of freedom (DF), and Least Significant Difference (LSD) for estimated marginal means. Missing data are handled within the linear mixed-effects regression model.

**Table S6** *Pain Intensity Trends Adjusted^†^ by Linear Mixed-Effects Regression Model.*

| Measure | Treatment Frequency | Time | m | Adjusted  Mean | SE | DF | 95 % CI | Change | | Cohen’s d | | p-value | |
| --- | --- | --- | --- | --- | --- | --- | --- | --- | --- | --- | --- | --- | --- |
| Pain Intensity VAS Scores | All Health Issues,  1+ Treatments | Pre-treatment | 144 | 6.15 | 0.21 | 256 | 5.72 – 6.55 |  |  |  |  |  |  |
|  |  | 3-month follow up | 90 | 4.02 | 0.26 | 292 | 3.52 – 4.53 | 3-month –  Pre-treatment | -2.13 | -0.85 | Large | <.001 | *** |
|  |  | 12-month follow up | 61 | 3.16 | 0.31 | 292 | 2.56 – 3.76 | 12-month –  3-month | -0.86 | -0.34 | Small | .014 | * |
|  |  |  |  |  |  |  |  | 12-month – Pre-treatment | -2.99 | -1.19 | Large | <.001 | *** |
|  | 1–5 Treatments | Pre-treatment | 31 | 6.27 | 0.45 | 251 | 5.39 – 7.15 |  |  |  |  |  |  |
|  |  | 3-months follow up | 21 | 4.98 | 0.53 | 286 | 3.94 – 6.02 | 3-months –  Pre-treatment | -1.30 | -0.52 | Moderate | .026 | * |
|  |  | 12-month follow up | 13 | 4.73 | 0.65 | 289 | 3.46 – 6.00 | 12-month –  3-month | -0.25 | -0.10 | No change | .727 | x |
|  |  |  |  |  |  |  |  | 12-month – Pre-treatment | -1.54 | -0.62 | Moderate | .024 | * |
|  | 6+ Treatments | Pre-treatment | 113 | 6.10 | 0.23 | 250 | 5.64 – 6.56 |  |  |  |  |  |  |
|  |  | 3-month follow up | 69 | 3.75 | 0.29 | 289 | 3.18 – 4.32 | 3-month –  Pre-treatment | -2.36 | -0.94 | Large | <.001 | *** |
|  |  | 12-month follow up | 48 | 2.72 | 0.34 | 289 | 2.05 – 3.39 | 12-month –  3-month | -1.03 | -0.41 | Small | .008 | ** |
|  |  |  |  |  |  |  |  | 12-month – Pre-treatment | -3.39 | -1.35 | Large | <.001 | *** |
|  |  |  |  |  |  |  |  |  | |  | |  | |
| SF-36 Bodily Pain Scores | Treatment Group | Time | n | Adjusted  Mean | SE |  | 95 % CI | Change | | Cohen’s d | | p-value | |
|  | All Patients, 1+ Treatments | Pre-treatment | 60 | 41.44 | 1.37 | 104 | 38.73 – 44.15 |  |  |  |  |  |  |
|  |  | 3-month follow up | 34 | 46.15 | 1.65 | 118 | 42.89 – 49.42 | 3-month –  Pre-treatment | 4.71 | 0.47 | Small | .004 | ** |
|  |  | 12-month follow up | 27 | 47.07 | 1.83 | 118 | 43.45 – 50.70 | 12-month –  3-month | 0.92 | 0.09 | No change | .644 | x |
|  |  |  |  |  |  |  |  | 12-month – Pre-treatment | 5.63 | 0.56 | Moderate | .004 | ** |
|  | 1–5 Treatments | Pre-treatment | 14 | 40.23 | 2.79 | 101 | 34.70 – 45.76 |  |  |  |  |  |  |
|  |  | 3-month follow up | 10 | 41.46 | 3.12 | 115 | 35.28 – 47.65 | 3-month –  Pre-treatment | 1.23 | 0.12 | No change | .688 | x |
|  |  | 12-month follow up | 5 | 41.72 | 3.93 | 111 | 33.94 – 49.51 | 12-month –  3-month | 0.26 | 0.03 | No change | .947 | x |
|  |  |  |  |  |  |  |  | 12-month – Pre-treatment | 1.49 | 0.15 | No change | .689 | x |
|  | 6+ Treatments | Pre-treatment | 46 | 41.78 | 1.54 | 102 | 38.72 – 44.84 |  |  |  |  |  |  |
|  |  | 3-month follow up | 24 | 47.77 | 1.90 | 115 | 44.01 – 51.53 | 3-month –  Pre-treatment | 5.99 | 0.60 | Moderate | .001 | ** |
|  |  | 12-month follow up | 22 | 48.57 | 2.03 | 115 | 44.56 – 52.59 | 12-month –  3-month | 0.81 | 0.08 | No change | .720 | x |
|  |  |  |  |  |  |  |  | 12-month – Pre-treatment | 6.80 | 0.68 | Moderate | .002 | ** |

**Effect size, Cohen’s d:** 0.00-0.19 = No change, 0.20-0.49 = Small, 0.50-0.79 = Moderate, ≥0.80 = Large. Negative Cohen’s d for VAS scores indicates a decrease in perceived pain intensity. Positive Cohen’s d for SF-36 Bodily Pain indicates an improvement in how pain affects the patient's quality of life, including the patient’s enhanced ability to perform daily activities.
**VAS Trends**: Treatment goals of achieving scores ≤ 3 are reached (p < .001).
**SF-36 Bodily Pain Trends**: Treatment goals of achieving BP scores ≥ 47 indicative of normal healthy population status, are reached (p < .01). **Treatment Frequency Impact**: Participants receiving six or more treatments demonstrate substantial improvements at 12 months (p < .001), whereas those receiving 1-5 treatment show a weaker response for VAS (p < .05) and a non-significant improvement in BP.
**Counts**: 'm' indicates the count of health issues, and 'n' indicates the number of patients.
**Significance Levels** from Linear Mixed Effect Regression between pre-treatment, 3- and 12-months follow-up are denoted as *** for p < .001, ** for p < .01, and * p < .05, while ‘x’ indicates no significant difference at p < .05.
**Model Details**^†^**:** In the linear mixed-effects regression model, means are adjusted for multiple observations from the same patient, the fixed effects are the intercept, time, number of treatments (1-5, 6+), and their interaction with time. Random effects are patient-specific intercepts, accounting for individual baseline variability. The model uses maximum likelihood estimation, Satterthwaite approximation for degrees of freedom (DF), and Least Significant Difference (LSD) for estimated marginal means. Missing data are handled within the linear mixed-effects regression model.

**Table S7** *SF-36 Physical and Mental Summary Score Trends Adjusted*^†^ *by Linear Mixed-Effects Regression Model.*

| Measure | Treatment Frequency | Time | n | Adjusted  Mean | SE | DF | 95 % CI | Change | | Cohen’s d | | p-value | |
| --- | --- | --- | --- | --- | --- | --- | --- | --- | --- | --- | --- | --- | --- |
| SF-36 Physical Component Summary Scores | All Patients, 1+ Treatments | Pre-treatment | 60 | 38.01 | 1.38 | 94 | 35.26 – 40.75 |  |  |  |  |  |  |
|  |  | 3-month follow up | 34 | 43.55 | 1.58 | 118 | 40.43 – 46.68 | 3-month –  Pre-treatment | 5.55 | 0.56 | Moderate | <.001 | *** |
|  |  | 12-month follow up | 27 | 46.01 | 1.73 | 118 | 42.59 – 49.43 | 12-month –  3-month | 2.46 | 0.25 | Small | .142 | x |
|  |  |  |  |  |  |  |  | 12-month –  Pre-treatment | 8.00 | 0.80 | Large | <.001 | *** |
|  | 1–5 Treatments | Pre-treatment | 14 | 37.39 | 2.85 | 92 | 31.73 – 43.06 |  |  |  |  |  |  |
|  |  | 3-month follow up | 10 | 40.14 | 3.09 | 109 | 34.02 – 46.25 | 3-month –  Pre-treatment | 2.74 | 0.27 | Small | .283 | x |
|  |  | 12-month follow up | 5 | 41.91 | 3.64 | 115 | 34.70 – 49.11 | 12-month –  3-month | 1.77 | 0.18 | No change | .580 | x |
|  |  |  |  |  |  |  |  | 12-month –  Pre-treatment | 4.51 | 0.45 | Small | .140 | x |
|  | 6+ Treatments | Pre-treatment | 46 | 38.14 | 1.58 | 94 | 35.01 – 41.27 |  |  |  |  |  |  |
|  |  | 3-month follow up | 24 | 44.63 | 1.82 | 115 | 41.03 – 48.23 | 3-month –  Pre-treatment | 6.49 | 0.65 | Moderate | <.001 | *** |
|  |  | 12-month follow up | 22 | 47.27 | 1.94 | 115 | 43.44 – 51.11 | 12-month –  3-month | 2.64 | 0.26 | Small | .163 | x |
|  |  |  |  |  |  |  |  | 12-month –  Pre-treatment | 9.13 | 0.91 | Large | <.001 | *** |
|  |  |  |  |  |  |  |  |  |  |  |  |  |  |
|  |  |  |  |  |  |  |  |  |  |  |  |  |  |
| SF-36 Mental Component Summary Scores | All Patients, 1+ Treatments | Pre-treatment | 60 | 41.04 | 1.22 | 98 | 38.62 – 43.47 |  |  |  |  |  |  |
|  |  | 3-month follow up | 34 | 46.26 | 1.44 | 118 | 43.40 – 49.12 | 3-month –  Pre-treatment | 5.22 | 0.52 | Moderate | <.001 | *** |
|  |  | 12-month follow up | 27 | 48.41 | 1.60 | 118 | 45.25 – 51.57 | 12-month –  3-month | 2.15 | 0.22 | Small | .198 | x |
|  |  |  |  |  |  |  |  | 12-month –  Pre-treatment | 7.37 | 0.74 | Moderate | <.001 | *** |
|  | 1–5 Treatments | Pre-treatment | 14 | 41.53 | 2.52 | 97 | 36.52 – 46.53 |  |  |  |  |  |  |
|  |  | 3-month follow up | 10 | 47.40 | 2.79 | 114 | 41.87 – 52.93 | 3-month –  Pre-treatment | 5.88 | 0.59 | Moderate | .027 | * |
|  |  | 12-month follow up | 5 | 46.52 | 3.43 | 114 | 39.72 – 53.32 | 12-month –  3-month | -0.88 | -0.09 | No change | .790 | x |
|  |  |  |  |  |  |  |  | 12-month –  Pre-treatment | 4.99 | 0.45 | Small | .115 | x |
|  | 6+ Treatments | Pre-treatment | 46 | 40.75 | 1.40 | 115 | 37.98 – 43.52 |  |  |  |  |  |  |
|  |  | 3-month follow up | 24 | 45.74 | 1.68 | 115 | 42.41 – 49.06 | 3-month –  Pre-treatment | 4.98 | 0.50 | Moderate | .001 | ** |
|  |  | 12-month follow up | 22 | 48.95 | 1.79 | 115 | 45.40 – 52.50 | 12-month –  3-month | 3.21 | 0.32 | Small | .096 | x |
|  |  |  |  |  |  |  |  | 12-month –  Pre-treatment | 8.20 | 0.82 | Large | <.001 | *** |

**Effect size, Cohen’s d:** 0.00-0.19 = No change, 0.20-0.49 = Small, 0.50-0.79 = Moderate, ≥0.80 = Large. A positive Cohen’s d for the SF-36 Physical and Mental Component Summaries suggests improvements in patients' physical functioning, emotional well-being, and social functioning, reflecting an overall enhancement in quality of life.
**Physical and Mental Component Summaries** **Trends**: Treatment goals of achieving scores ≥ 47, indicative of normal health, are reached (large and moderate effect size, p <.001).
**Counts**: 'n' indicates the number of patients.
**Treatment Frequency Impact:** Participants receiving six or more treatments demonstrate substantial improvements at 12 months for PCS and MCS (large effect size, p <.001), whereas those receiving 1-5 treatment shows a weaker response.
**Significance Levels** from Linear Mixed Effect Regression between pre-treatment, 3- and 12-months follow-up are denoted as *** for p < .001, ** for p < .01, and * p < .05, while ‘x’ indicates no significant difference at p < .05.
**Model Details**^†^**:** In the linear mixed-effects regression model, means are adjusted for multiple observations from the same patient, the fixed effects are the intercept, time, number of treatments (1-5, 6+), and their interaction with time. Random effects are patient-specific intercepts, accounting for individual baseline variability. The model uses maximum likelihood estimation, Satterthwaite approximation for degrees of freedom (DF), and Least Significant Difference (LSD) for estimated marginal means. Missing data are handled within the linear mixed-effects regression model.

**Table S8** *Proportion of Patients Experiencing Significant Improvements in Physical Health, Across the Full Dataset and Propensity Score Matched Dataset, Comparing Proportions of Patients Improved Versus Worsened.*

| Measure | Treatment Frequency | Outcome Category | Frequency | Sample Size | Proportion | SE | Percentage Responders | p-value | |
| --- | --- | --- | --- | --- | --- | --- | --- | --- | --- |
| SF-36  Physical Component Summary Scores  (Full Dataset) | All Patients, 1+ Treatments | Improved to ≥ 47 | 16 | 32 | 0.50 | n.a. | 50% Complete cures |  |  |
|  |  | Improved to ≥ 47  or 3+ T-scores | 23 | 32 | 0.72 | 0.09 | 72% Responders | <.001 | *** |
|  |  | Improved to ≥ 47  or 8+ T-scores | 23 | 32 | 0.72 | n.a. | 72% Strong responders |  |  |
|  |  |  |  |  |  |  |  |  |  |
|  |  | Unchanged Normal Physical Health | 1 | 32 | 0.03 | 0.17 |  |  |  |
|  |  |  |  |  |  |  |  |  |  |
|  |  | Unchanged Below Norm | 7 | 32 | 0.22 | 0.16 |  |  |  |
|  |  |  |  |  |  |  |  |  |  |
|  |  | Worsened 3+ T-scores | 1 | 32 | 0.03 | 0.17 |  |  |  |
|  | 1–5 Treatments | Improved to ≥ 47 | 2 | 8 | 0.25 | n.a. | 25% Complete cures |  |  |
|  |  | Improved to ≥ 47  or 3+ T-scores | 5 | 8 | 0.63 | 0.22 | 63% Responders | 0.102 | x |
|  |  | Improved to ≥ 47  or 8+ T-scores | 5 | 8 | 0.63 | n.a. | 63% Strong Responders |  |  |
|  |  |  |  |  |  |  |  |  |  |
|  |  | Unchanged Normal Physical Health | 0 | 8 | 0.00 | - |  |  |  |
|  |  |  |  |  |  |  |  |  |  |
|  |  | Unchanged Below Norm | 2 | 8 | 0.25 | 0.31 |  |  |  |
|  |  | Worsened 3+ T-scores | 1 | 8 | 0.13 | 0.33 |  |  |  |
|  | 6+ Treatments | Improved to ≥ 47 | 14 | 24 | 0.58 | n.a. | 58% Complete cures |  |  |
|  |  | Improved to ≥ 47  or 3+ T-scores | 18 | 24 | 0.75 | 0.10 | 75% Responders | <.001 | *** |
|  |  | Improved to ≥ 47  or 8+ T-scores | 18 | 24 | 0.75 | n.a. | 75% Strong responders |  |  |
|  |  |  |  |  |  |  |  |  |  |
|  |  | Unchanged Normal Physical Health | 1 | 24 | 0.04 | 0.20 |  |  |  |
|  |  |  |  |  |  |  |  |  |  |
|  |  | Unchanged Below Norm | 5 | 24 | 0.21 | 0.18 |  |  |  |
|  |  |  |  |  |  |  |  |  |  |
|  |  | Worsened 3+ T-scores | 0 | 24 | 0.00 | - |  |  |  |
| SF-36  Physical Component Summary Scores  (Propensity Score Matched Dataset) | All Patients, 1+ Treatments | Improved to ≥ 47 | 6 | 14 | 0.43 | n.a. | 43% Complete cures |  |  |
|  |  | Improved to ≥ 47  or 3+ T-scores | 9 | 14 | 0.64 | 0.16 | 64% Responders | 0.011 | * |
|  |  | Improved to ≥ 47  or 8+ T-scores | 9 | 14 | 0.64 | n.a. | 64% Strong responders |  |  |
|  |  |  |  |  |  |  |  |  |  |
|  |  | Unchanged Normal Physical Health | 0 | 14 | - | - |  |  |  |
|  |  |  |  |  |  |  |  |  |  |
|  |  | Unchanged Below Norm | 4 | 14 | 0.29 | 0.23 |  |  |  |
|  |  |  |  |  |  |  |  |  |  |
|  |  | Worsened 3+ T-scores | 1 | 14 | 0.07 | 0.26 |  |  |  |
|  | 1–5 Treatments | Improved to ≥ 47 | 2 | 7 | 0.29 | n.a. | 29% Complete cures |  |  |
|  |  | Improved to ≥ 47  or 3+ T-scores | 4 | 7 | 0.57 | 0.25 | 57% Responders | 0.180 | x |
|  |  | Improved to ≥ 47  or 8+ T-scores | 4 | 7 | 0.57 | n.a. | 57% Strong responders |  |  |
|  |  |  |  |  |  |  |  |  |  |
|  |  | Unchanged Normal Physical Health | 0 | 7 | 0.00 | - |  |  |  |
|  |  |  |  |  |  |  |  |  |  |
|  |  | Unchanged Below Norm | 2 | 7 | 0.29 | 0.32 |  |  |  |
|  |  |  |  |  |  |  |  |  |  |
|  |  | Worsened 3+ T-scores | 1 | 7 | 0.14 | 0.35 |  |  |  |
|  | 6+ Treatments | Improved to ≥ 47 | 4 | 7 | 0.57 | n.a. | 57% Complete cures |  |  |
|  |  | Improved to ≥ 47  or 3+ T-scores | 5 | 7 | 0.71 | 0.20 | 71% Responders | 0.014 | * |
|  |  | Improved to ≥ 47  or 8+ T-scores | 5 | 7 | 0.71 | 0.20 | 71% Strong responders |  |  |
|  |  |  |  |  |  |  |  |  |  |
|  |  | Unchanged Normal Physical Health | 0 | 7 | - | - |  |  |  |
|  |  |  |  |  |  |  |  |  |  |
|  |  | Unchanged Below Norm | 2 | 7 | 0.29 | 0.32 |  |  |  |
|  |  |  |  |  |  |  |  |  |  |
|  |  | Worsened 3+ T-scores | 0 | 7 | - | - |  |  |  |

**n.a***.*: Standard Error (SE) is not applicable for subgroups of Responders.
**Health Status Changes:** A two-sided McNemar test compared post-treatment changes in physical health. The analysis focuses on the proportions of patients showing improved versus worsened outcomes (discordant pairs), assuming that improvements and deteriorations are equally likely without treatment. Significantly more patients showed improvement than worsening, with 72% of patients improved versus 3% worsening (Difference in Proportions = 0.69, Standard Error = 0.09, *Z* = 4.49, p < 0.001). Furthermore, 50% regained normal physical health (PCS ≥ 47), indicating complete cures. Overall, 72% were strong responders, while 28% did not improve. In the propensity-matched dataset, 64% were responders, 43% complete cures. As the proportion of responders did not differ significantly between the full dataset and the smaller propensity score-matched dataset (Chi-squared test χ² = 0.29, DF = 1, p = 0.59), this strongly supports the robustness of the results.
**Treatment Frequency Impact:** More treatments consistently resulted in a higher proportion of patients experiencing significant improvements in physical health and achieving "complete cures," reaching the Norwegian norm.
**Significance Levels from McNemar test,** comparing proportions of patients improved versus worsened, are denoted as *** for p < .001, ** for p < .01, and * p < .05, while 'x' indicates no significant difference at p < .05. Patients with missing data were excluded from this analysis, as the McNemar test requires complete pairs. Of 75 patients, 15 did not submit pre-treatment SF-36 forms and 43 did not submit post-treatment SF-36 forms, leaving 32 pairs for the McNemar test. For the propensity score-matched dataset, there were no missing data, retaining 14 pairs for analysis.
Proportions of responders were similar in full and propensity-matched datasets (Chi-square test, p=.59), supporting the robustness of the findings.
**Impact of pre-treatment physical health status (Full Dataset):** Patients were categorized into three PCS groups: very poor (PCS < 30), poor (PCS 30-39), and slightly decreased health (PCS 40-47). Similar proportions of responders were found across all groups (Chi-squared test χ² = 2.2, DF = 2, p = .34), indicating a generally positive treatment effect. Fewer patients with poor or very poor pre-treatment health achieved complete cures compared to those with slightly decreased health, (Chi-squared test χ² =10.4, DF = 2, p = 0.004).

**Figure S1**

*Effect size and sample size relationship with statistical power. (A) Graph showing the estimated power by sample size for different effect sizes. (B) Table with effect size categories, desired power, significance level, and corresponding required sample sizes..*


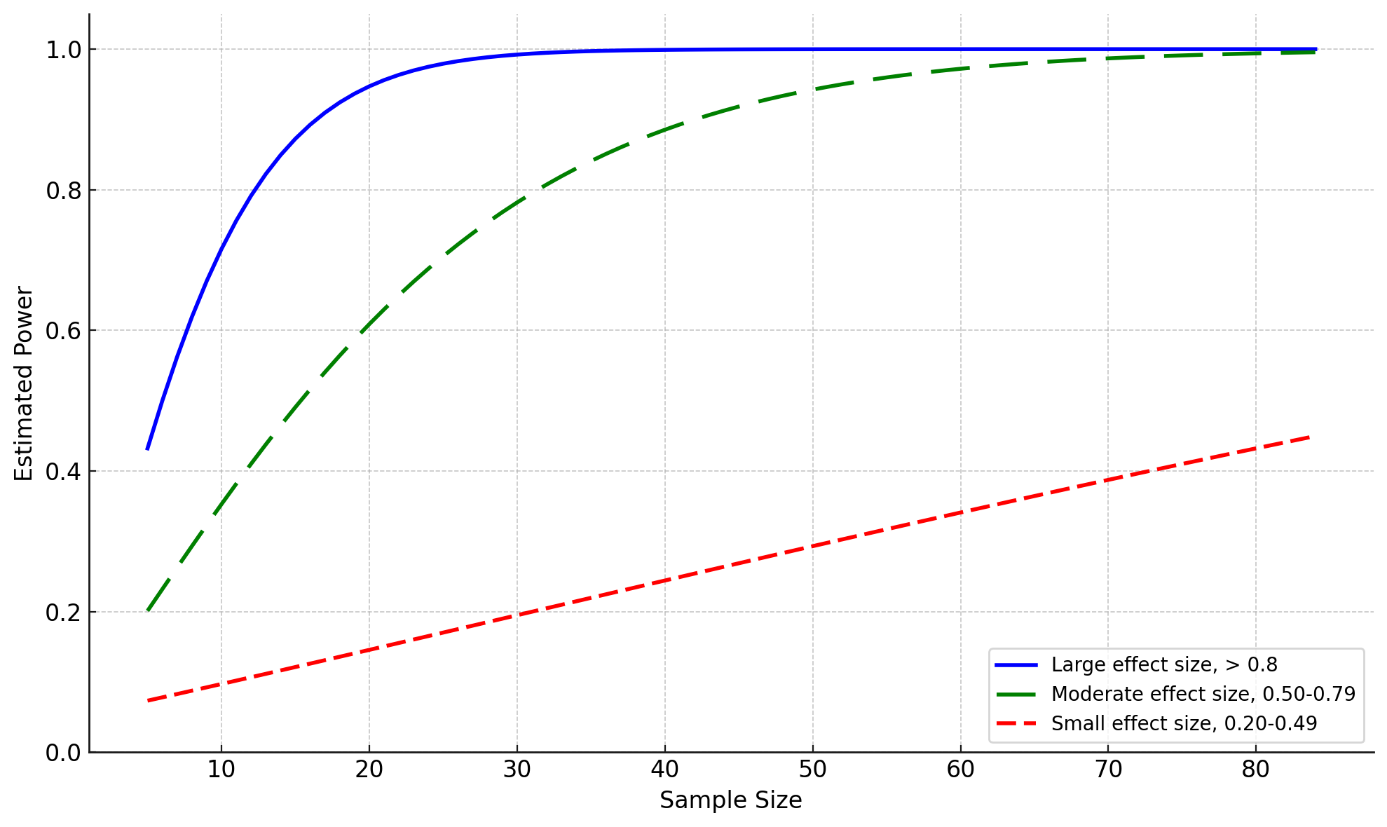


(B)

(A)

| Effect Size | Desired Power  (1-β) | Significance  Level (α) | Desired  Sample Size |
| --- | --- | --- | --- |
| Large, >0.8 | 0.8 | 0.05 | 15 |
| Moderate, 0.50-0.79 | 0.8 | 0.05 | 34 |
| Small, 0.20-0.49 | 0.8 | 0.05 | 199 |

**Note:** Our power analysis indicates that the full dataset (n=75) was more than sufficient to detect large effect sizes (>0.8) in changes in PCS scores**,** with 80% power at α=0.05. (Cohen's d=0.8, 95% CI: 0.4-1.2, power 0.99).
The matched dataset analysis (n=7 pairs, 14 patients) revealed a moderate effect size (Cohen's d=0.5, 95% CI: 0.1-1.1), achieving 54% power while maintaining statistical significance (p=0.04). Although underpowered by conventional standards for moderate effects, the matched analysis yielded results consistent with those from the full dataset.
Based on power analysis, a sample size of 34 patients (17 pairs) would be required to achieve 80% power for detecting an effect size of 0.5. Despite sample size limitations in the matched analysis, the consistent direction of effect and statistical significance support the reliability of our findings, though confirmation in larger studies is warranted.

**Checklist S1**

**STRICTA 2010 Checklist of information to include when reporting interventions in an acupuncture study.**

| Item | Recommendation | Relevant details from manuscript | Reported on section | Page |
| --- | --- | --- | --- | --- |
| 1. Acupuncture rationale |  |  |  |  |
|  | Style of acupuncture (e.g. Traditional Chinese Medicine, Japanese, Korean, Western medical, Five Element, ear acupuncture, etc) | Traditional Chinese Medicine (TCM) principles with qi-arrival (De-Qi) techniques. | Background | 4 |
|  | Reasoning for treatment provided, based on historical context, literature sources, and/or consensus methods, with references where appropriate | Historical context and literature supporting qi-arrival acupuncture's efficacy in chronic pain management. | Background | 4 |
|  | Extent to which treatment was varied | Number of treatments and points selected were individualized based on patient needs and treatment response. | Methods | 8 |
| 1. Details of needling |  |  |  |  |
|  | Number of needle insertions per subject per session (mean and range where relevant) | Individualized based on patient needs and treatment response. | Methods | 8 |
|  | Names (or location if no standard name) of points used (uni/bilateral) | Standardized points and practitioner-selected points used (bilateral). | Methods. Table S2. | 8 |
|  | Depth of insertion, based on a specified unit of measurement, or on a particular tissue level | Needles inserted to achieve qi-arrival, varying depths depending on point. | Methods | 8 |
|  | Response sought (e.g. *de qi* or muscle twitch response) | Qi-arrival (de qi sensation) sought by brief manual manipulation. | Methods | 8 |
|  | Needle stimulation (e.g. manual, electrical) | Manual stimulation used to achieve desired sensation. | Methods | 8 |
|  | Needle retention time | Sessions lasted 45-60 minutes; first session 60-90 minutes. | Methods | 8 |
|  | Needle type (diameter, length, and manufacturer or material) | Diameter 0.20 mm, length 40 mm, Green Nature, manufactured by Suzhou ZhongJing Life & Science Technology Ltd, Wujiang, China | Methods | 8 |
| 1. Treatment regimen |  |  |  |  |
|  | Number of treatment sessions | Median 6 (range 1-27), Mean 7.3, SD 4.6. | Results. Table 1 | 10 |
|  | Frequency and duration of treatment sessions | Treatment frequency tailored to patient need; each session lasted 45-60 minutes. | Table 1 |  |
| 1. Other components of treatment |  |  |  |  |
|  | Details of other interventions administered to the acupuncture group (e.g. moxibustion, cupping, herbs, exercises, lifestyle advice) | No other interventions administered. | - | - |
|  | Setting and context of treatment, including instructions to practitioners, and information and explanations to patients | Treatment was conducted in the outpatient family medicine practice of Dr. med. Jan Baak AS, Tananger, Norway. All procedures were administered by Dr. med. Jan Baak, a Western-trained MD with dual qualifications as a general practitioner and TCM specialist. Patients received standardized information about acupuncture for chronic pain. | Method | 6 |
| 1. Practitioner background |  |  |  |  |
|  | Description of participating acupuncturists (qualification or professional affiliation, years in acupuncture practice, other relevant experience) | Experienced family medicine practitioner since 1974, pathologist since 1980, acupuncture 1-year full time training at Shanghai University of Traditional Chinese Medicine 2008-2009, TCM specialisation Technical University Munich 2012-2016; medical practice in combined family medicine and acupuncture since 2013. | Method | 7 |
| 1. Control or comparator interventions |  |  |  |  |
|  | Rationale for the control or comparator in the context of the research question, with sources that justify this choice | Different treatment frequencies (1-5 sessions vs. 6+ sessions) were used to explore the dose-response relationship of acupuncture in chronic pain management. This comparison was based on established research showing that treatment frequency significantly impacts clinical outcomes. White et al.[26] demonstrated that varying treatment numbers affects outcomes, confirming a clear dose-response relationship in acupuncture therapy. Supporting this approach, Yang et al.[27] concluded that fewer than 6 sessions is considered insufficient for treating chronic low back pain, providing a clinically relevant threshold for our frequency groupings. This design allowed us to evaluate whether increased treatment frequency leads to proportionally improved patient outcomes while establishing minimum effective treatment parameters. | Method | 8 |
|  | Precise description of the control or comparator. If sham acupuncture or any other type of acupuncture-like control is used, provide details as for Items 1 to 3 above. | Propensity score matching was used to match patients from the 1-5 session and 6+ session groups based on demographic and baseline characteristics.  The only difference between groups was the number of treatment sessions. | Method | 9 |

Note: This checklist should be read in conjunction with the explanations of the STRICTA items.

It is designed to replace CONSORT 2010's item 5 when reporting an acupuncture trial.

**Checklist S2**

**STROBE Statement—Checklist of items that should be included in reports of observational studies.**

| **Section** | **Item No.** | **Recommendation** | **Page No.** | **Relevant text from manuscript** |
| --- | --- | --- | --- | --- |
| **Title and abstract** |  |  |  |  |
|  | 1(a) | Indicate the study's design with a commonly used term in the title or the abstract | 1 | Article type: Observational study |
|  | 1(b) | Provide in the abstract an informative and balanced summary of what was done and what was found | 2 | Patients with chronic pain unresponsive to conventional treatments received acupuncture in a real-world clinical setting. After treatment, 72% of patients experienced clinically significant improvements, with 50% achieving complete recovery to normal Norwegian health standards. Propensity score matching suggested these findings were not due to baseline differences. |
| **Introduction** |  |  |  |  |
| Background/rationale | 2 | Explain the scientific background and rationale for the investigation being reported | 3 | Chronic pain is a frequent disease with higher prevalence among 65+ persons. In TCM theory, the therapeutic effect of acupuncture is closely related to the achievement of 'De-Qi' (obtaining qi), a specific sensation considered essential for clinical efficacy in traditional acupuncture practice |
| Objectives | 3 | State specific objectives, including any prespecified hypotheses | 3,6 | We conducted a prospective observational study to assess the long-term therapeutic efficacy of acupuncture in chronic pain patients unresponsive to conventional treatments. We also introduced a novel statistical approach to evaluate acupuncture success by quantifying the proportion of patients who regained normal health according to Norwegian national health standards. Additionally, we applied the Bradford Hill criteria to assess whether the relationship between De-Qi acupuncture and observed health improvements was causal rather than merely associative |
| Methods Study design | 4 | Present key elements of study design early in the paper | 6 | The study is a non-interventional study also an observational study |
| Methods Setting | 5 | Describe the setting, locations, and relevant dates, including periods of recruitment, exposure, follow-up, and data collection | 6 | Enrolment: All consecutive new patients in a first-line medical practice 2015-2017, with 12 months follow up. Treatments were administered in a family medical practice setting by an experienced western-trained medical doctor with formal acupuncture training. |
| Methods Participants | 6(a) | Give the eligibility criteria, and the sources and methods of selection of participants. Describe methods of follow-up | 7 | Specific inclusion criteria: (1) chronic pain defined as persistent or recurrent pain lasting for at least 3 months; (2) pain located in the neck, shoulder, back, hip, or knee regions identified by ICD-11 codes; (3) previous unsuccessful treatments with conventional therapies; and (4) ability to complete self-assessment questionnaires. Patients self-reported their health problems and quality of life by completing paper-based surveys. |
| Methods Participants | 6(b) | For matched studies, give matching criteria and number of exposed and unexposed | 9 | Propensity score matching (PSM) was used to reduce possible selection bias. Patients were matched 1:1 using a greedy matching algorithm, with matching quality assessed by p-values > 0.05 for baseline characteristics and standardized mean differences < 0.1 for propensity scores. This procedure matched n=7 patients with 6+ treatments (exposed group) with n=7 patients receiving 1-5 treatments (control group), creating well-balanced groups for analyses of dose-effect analysis. The matched cohort showed no significant differences in baseline characteristics between the treatment groups. |
| Methods Variables | 7 | Clearly define all outcomes, exposures, predictors, potential confounders, and effect modifiers. Give diagnostic criteria, if applicable | 10 | Pain intensity and quality of life were assessed using VAS and the SF-36 Health Survey. |
| Methods Data sources/measurement | 8* | For each variable of interest, give sources of data and details of methods of assessment (measurement). Describe comparability of assessment methods if there is more than one group | 9-11 | Self-reported pain intensity and quality of life were assessed using VAS and the SF-36 Health Survey at pre-treatment, 3 and 12 months. |
| Methods Bias | 9 | Describe any efforts to address potential sources of bias | 9 | Propensity score matching (PSM) was used to reduce possible selection bias. The treatment doctor was unaware of the patient’s enrolment status to reduce treatment bias. |
| Methods Study size | 10 | Explain how the study size was arrived at | 9 | Out of 354 potential participants, 75 chronic pain patients (fulfilling the criteria in 6a) were included in the study, and 14 (7 pairs) in the propensity score matched subgroup. A pre-study power calculation indicated that 34 patients would be needed to detect a large effect size (Cohen's d=0.8) with 80% power at α=0.05. For the primary outcome analysis, our sample of 75 patients exceeded this requirement. Post-hoc power analysis confirmed that the observed effect size of 0.8 (large effect) was detected with 99% power (p<0.05). For the propensity-matched dataset (n=14), despite the smaller sample size, results remained statistically significant (p<0.05) with meaningful effect sizes comparable to those observed in the full cohort. This consistency between the full and matched analyses strengthens confidence in our findings, as the matched analysis controlled for potential confounding while producing significant results that aligned with the primary analysis. |
| Methods Quantitative variables | 11 | Explain how quantitative variables were handled in the analyses. If applicable, describe which groupings were chosen and why | 8-10 | Patients were divided into two age groups to test how elderly patients responded to treatment. Patients were divided into two treatment frequency groups to test the impact of treatment frequency. Patients were divided into three pre-treatment PCS scores categories to test how patients with poor initial health responded to treatment. |
| Methods Statistical methods | 12 | Describe all statistical methods, including those used to control for confounding | 9 | Statistical analyses conducted using IBM SPSS v29. Trends were assessed by linear mixed effect regression. Mcnemar test compared improved vs worsened. Chi squared test was used to test response rates/cure rates and to compare results for subgroups. Cohens d was used to assess effect size. We applied Hill’s 9 criteria to assess causation. |
| Results Participants | 13* | Report numbers of individuals at each stage of study | Figure1 | Out of 354 potential participants, 101 were enrolled with consent, 75 met inclusion criteria for chronic pain and were included for analysis.  3-month Follow-up: Completed follow-up (n = 34, m = 90) Lost to follow-up (n = 41): registration forms not submitted.  12-month Follow-up: Completed follow-up (n = 27, m= 61) Lost to follow-up (n = 48): registration forms not submitted. |
| Results Descriptive data | 14* | Give characteristics of study participants | 10  Table 1 | 75 patients 158 ICD-coded pain issues Age 20-79 (median 50), 65% women |
| Results Outcome data | 15* | Report numbers of outcome events or summary measures over time | 10-11  Table S5 | VAS, SF-36, responder rates, complete cures. |
| Results Main results | 16 | Give unadjusted estimates and, if applicable, confounder-adjusted estimates and their precision | 11  Table S5-S7 | Descriptive statistics are provided in supplementary table S5. Results from linear mixed effect regression is adjusted for multiple measurements for a patient. Average VAS pain scores decreased from 6.2 pre-treatment to 4.0 at 3 months and 3.2 at 12 months. 72% of participants were strong responders 50% achieved 'complete cures' |
| Results Other analyses | 17 | Report other analyses done—e.g., analyses of subgroups and interactions, and sensitivity analyses | 11  Table S8 | Similar proportions of responders were found across all pre-treatment health groups.  Similar proportions of responders were found in the propensity score matched dataset as in the entire cohort. |
| Discussion Key results | 18 | Summarise key results with reference to study objectives | 12 | In this observational study, 72% of patients with chronic pain were associated with clinically significant improvements, with many achieving Norwegian normal health standards. Propensity score analysis strengthened these associations while controlling for confounders. |
| Discussion Limitations | 19 | Discuss limitations of the study, taking into account sources of potential bias or imprecision. Discuss both direction and magnitude of any potential bias | 13 | While our observational real-world study shows substantial improvements in patients with chronic pain, we acknowledge inherent limitations. Without a randomized control group, we cannot definitively rule out the influence of natural history, regression to the mean, or non-specific effects of treatment. However, given the chronic nature and treatment-resistant history of our patients' conditions (median duration 18 months), the substantial improvements observed warrant further investigation. |
| Discussion Interpretation | 20 | Give a cautious overall interpretation of results considering objectives, limitations, multiplicity of analyses, results from similar studies, and other relevant evidence | 12 | The effect sizes observed in our study are comparable to those reported in other acupuncture studies for chronic pain. While the Hill criteria analysis supports a potential causal relationship, we acknowledge the limitations of our observational design. Our findings suggest a minimum of 6 treatment sessions may be associated with better outcomes, including in elderly patients and those with very poor initial health. |
| Discussion Generalisability | 21 | Discuss the generalisability (external validity) of the study results | 12 | Findings point to potentially much less personal suffering and huge cost savings if medical acupuncture is adopted as a routine treatment in therapy-resistant given-up chronic pain patients |
| Other information Funding | 22 | Give the source of funding and the role of the funders for the present study and, if applicable, for the original study on which the present article is based | 17 | Financial support VL: None to declare. JB: Jan Baak, MD was owner of Dr. med. Jan Baak AS, Tananger, Norway. Retired on January 1, 2024 |

*Give information separately for exposed and unexposed groups.

**Note:** An Explanation and Elaboration article discusses each checklist item and gives methodological background and published examples of transparent reporting. The STROBE checklist is best used in conjunction with this article (freely available on the Web sites of PLoS Medicine at [http://www.plosmedicine.org/,](http://www.plosmedicine.org/) Annals of Internal Medicine at [http://www.annals.org/,](http://www.annals.org/) and Epidemiology at [http://www.epidem.com/).](http://www.epidem.com/)) Information on the STROBE Initiative is available at [http://www.strobe-statement.org.](http://www.strobe-statement.org/)
